# Genome-wide characterization of soybean *RALF* genes and their expression responses to *Fusarium oxysporum*

**DOI:** 10.3389/fpls.2022.1006028

**Published:** 2022-10-06

**Authors:** Yuhan Liu, Yuhui Chen, Hengke Jiang, Zhaowei Shui, Yujun Zhong, Jing Shang, Hui Yang, Xin Sun, Junbo Du

**Affiliations:** ^1^ College of Agronomy, Sichuan Agricultural University, Chengdu, China; ^2^ Research Center for Modern Agriculture of the Middle East, Sichuan Agricultural University, Chengdu, China; ^3^ Key Laboratory of Crop Ecophysiology and Farming System in Southwest China, Ministry of Agriculture, Sichuan Agricultural University, Chengdu, China

**Keywords:** GmRALF, soybean immune, evolution, *Fusarium*, genome wide

## Abstract

RALFs (RAPID ALKALINIZATION FACTORs) are small peptides required for plant growth, development and immunity. RALF has recently been discovered to regulate plant resistance to fungal infection. However, little is known in crops, particularly in soybean. Here, 27 *RALFs* were identified in the genome of *Glycine max*. All *Glycine max RALFs* (*GmRALFs*) and 34 Arabidopsis *RALFs* were classified into 12 clades *via* the phylogenetic analyses. Gene structures, conserved motifs, chromosome distribution and *cis*-elements were analyzed in this study. Furthermore, 18 *GmRALFs* were found in response to *Fusarium oxysporum* (*F. oxysporum*) infection in soybean and to have distinct expression patterns. Among them, secretory function of two GmRALFs were identified, and three GmRALFs were detected to interact with FERONIA in *Glycine max* (GmFERONIA, GmFER). Our current study systematically identified and characterized *GmRALFs* in the soybean genome, laying a groundwork for further functional analyses and soybean breeding.

## Introduction

Plants, as sessile organisms, have evolved unique signaling systems to cope with increasingly complex environmental conditions. Plant small peptide hormones are essential for plant development and environmental responses. Since the discovery of systemin in tomato, a large number of plant small peptide hormones, such as phytosulfokines (PSK), CLAVATA3 (CLV3), hydroxyproline-rich glycopeptide systemin (HypSyc), tracheary element differentiation inhibitory factor (TDIF) and others, have been identified (Pearce et al., 1991; Fletcher et al., 1999; Pearce et al., 2001a; Ito et al., 2006). RALFs are small cysteine-rich secreted peptides discovered firstly in tobacco leaf extracts to induce rapid alkalization of tobacco cell suspension medium (Pearce et al., 2001b). RALF homologs are abundant in the plant kingdom and play an important role in plant reproductive growth, vegetative growth and immunity (Pearce et al., 2001b; Covey et al., 2010; Mingossi et al., 2010; Bergonci et al., 2014; Morato Do Canto et al., 2014; Stegmann et al., 2017).

It is well acknowledged that the primary structure of a protein determines its function. An Arg-Arg (RR) motif, commonly found in animals and yeast, was identified at the N-terminus of the tobacco RALF peptide, implying that this conserved dibasic site may be the cleavage site for the proteolytic enzyme separating the propeptide from the mature active RALF peptide (Pearce et al., 2001b). And this view was later confirmed (Matos et al., 2008; Srivastava et al., 2009). RALF22 and RALF23 are cleaved at this site by site-1 protease (S1P), a plant subtilisin-like serine protease, which then regulate salt tolerance and plant immunity respectively in Arabidopsis (Stegmann et al., 2017; Zhao et al., 2018).

The YISY motif, which is involved in receptor binding and alkalinization activity, is a crucial motif for RALF activity (Pearce et al., 2010). When the isoleucine is replaced by alanine, the activity of RALF peptide and its ability to inhibit root growth are significantly reduced (Pearce et al., 2010). Besides, the C-terminal RCRR(S) motif and the four cysteine residues are important for the activity of RALFs (Blackburn et al., 2020). The disulfide bonds formed by the four conserved cysteine play a key role in the three-dimensional conformation and biological activity of RALF peptides (Blackburn et al., 2020). The second disulfide bond of RALF peptide is required to inhibit root growth or flagellin peptide 22 (flg22)-induced ROS burst (Zhang et al., 2020). Interestingly, RALFs lacking these typically conserved motifs are widespread in plants, but their function remains largely unknown (Campbell and Turner, 2017). Unlike plants, RALFs in fungi and nematodes generally lack the first disulfide bond (Moussu et al., 2020; Zhang et al., 2020).

Previous studies found that *RALFs* exist in the genomes of dicotyledon, monocots and gymnosperms (Zhu et al., 2021). RALFs are important contributors to plant growth and development (Murphy and De Smet, 2014; Zhu et al., 2021). RALF peptides have the ability to inhibit cell expansion and growth (Murphy and De Smet, 2014; Blackburn et al., 2020; Zhu et al., 2021). This process is regulated by RALF-FERONIA (FER) pathway (Haruta et al., 2014). FER is a receptor for RALF, and when it binds to RALF, the downstream phosphorylation signaling cascade reaction will be activated, inhibiting plasma membrane H^+^-ATPase activity, increasing apoplastic pH, and finally reducing cell elongation (Haruta et al., 2014). Overexpression of *RALF23* in Arabidopsis resulted in similar phenotypes such as growth retardation and plant dwarfism (Srivastava et al., 2009). Artificially synthesized *RALF19*, *RALF22*, *RALF23*, *RALF24*, *RALF31*, *RALF33* and *RALF34* were also found to inhibit hypocotyl elongation (Murphy and De Smet, 2014). According to additional research, RALF significantly influenced the plant reproductive development. Maintaining pollen tube integrity necessitates RALF4 and RALF19, as well as their receptors Buddha’s Paper Seal (BUPS) 1 and BUPS2 (Ge et al., 2017). Similar to the *bups1 bups2* mutant, the Arabidopsis *ralf4 ralf19* mutation caused the pollen tube burst to occur immediately after pollen germination without affecting vegetative growth (Ge et al., 2017). In addition, RALF4 and RALF19 are required for BUPS1 to maintain cell wall integrity (Zhou et al., 2021). *RALF6*, *RALF7*, *RALF16*, *RALF36* and *RALF37* were found to regulate *Arabidopsis* fertilization by establishing the polytube block at the septum that prevent multiple pollen tubes outlets (Zhong et al., 2022). In addition to Arabidopsis, *RALFs* in other plants are also important for their growth and development. For tomatoes, *RALF* in *Solanum lycopersicum* (*SlpRALF*) also inhibited the elongation of the pollen tube (Covey et al., 2010). *ScRALF3* in *Solanum chacoense* participated in the communication between the sporophyte and the female gametophyte in a solanaceous species (Chevalier et al., 2013). *PtdRALF*, identified in hybrid poplar (*Populus trichocarpa* x *Populus deltoides*), was expressed in almost all tissues of poplar, and the expression level of *PtdRALF2* decreased obviously after being treated by Methyl Jasmonate (MeJA) (Haruta and Constabel, 2003).

Compared with growth and development, studies on RALF regulating immunity are rare. Upon cleavage by S1P, the mature RALF23 suppresses ROS bursts triggered by flg22 or diseases, by disrupting the complex formation of flagellin sensing 2 (FLS2) with its co-receptor BRASSINOSTEROID INSENSITIVE 1-ASSOCIATED RECEPTOR KINASE 1 (BAK1) (Stegmann et al., 2017). In addition, some microorganisms can also secrete RALF-like peptides to inhibit host immunity. For example, *Mi*RALF1 and *Mi*RALF3 proteins encoded by root-knot nematodes (RKNS) *Meloidogyne incognita* can promote the parasitism of nematodes through hijacking FER to inhibit plant immunity (Zhang et al., 2020; Zhang et al., 2021). Furthermore, *Fusarium* (F)-RALF protein secreted by *F. oxysporum* can induce alkalization and inhibit plant immune response to promote fungal toxicity (Masachis et al., 2016).

Soybean root rot is a severe and widespread soil-borne fungal disease that can occur during any growing period of soybean, causing death and seriously reducing soybean production (Chang et al., 2018; Xu and Wei, 2020). *Fusarium* spp., *Pythium* spp., *Phytophthora* spp. and *Rhizoctonia* spp. are the main pathogens of soybean root rot (Wrather et al., 1997; Fravel et al., 2003; Jiang et al., 2012; Liu et al., 2016; Chang et al., 2018). And *Fusarium* is the dominant pathogen of soybean root rot in many areas. The yield of soybean was seriously reduced by *Fusarium* root rot. (Arias et al., 2013). Nonetheless, the mechanisms underlying soybean immune response to *F. oxysporum* are still largely unknown.

In this study, we identified the *RALF* gene family in soybean genome and defined the expression patterns of the whole *GmRALF* family in response to *F. oxysporum* infection. In addition, five members were identified as being significant response to *F. oxysporum* infection, and their functions were preliminarily investigated. Our results demonstrated that there were 27 *GmRALFs* in soybean. All *GmRALFs* were divided into eight subfamilies by phylogenetic tree analysis. The majority of GmRALFs contained typical domains with the gene structure and motif composition analysis. Many stress response elements, such as defense response elements, were identified by analyzing the *cis*-elements in the promoter region of the *GmRALFs*. In response to *F. oxysporum* infection, 18 *GmRALFs* showed different expression patterns. *GmRALF4*, *GmRALF5*, *GmRALF10*, *GmRALF24* and *GmRALF25* showed the highest response. Moreover, GmRALF4 and GmRALF24 are secreted peptides. The extracellular domain of GmFER (GmFERed) can interact with GmRALF4, GmRALF10 and GmRALF24.

## Materials and methods

### Plant materials and growth conditions

The *Fusarium* susceptible soybean variety Jiuyuehuang identified by our previous studies was used as a material (Bawa et al., 2019). Soybean seeds with full and uniform size were selected and placed in a petri dish and sterilized with chlorine for 16 h (Chen et al., 2018). The sterilized seeds are densely planted in flowerpots containing moderately humid vermiculite and covered with tin foil. Soybean plants were grown under dark conditions at 25°C for 4 days. Add water every other day, relative humidity control at about 85%.

### Fungal strains, culture conditions and infection assays

The strain of *Fusarium* was cultured on potato dextrose agar (PDA, 200 g•L^-1^ potato, 15 g•L^-1^ agar and 10 g•L^-1^ glucose anhydrous) containing 50 µg·mL^−1^ streptomycin (Chang et al., 2020).

A 7-day-old *F. oxysporum* block with a diameter of 1.5 cm was drilled with a perforator and inoculated into the hypocotyl of soybean seedlings (the inoculation site was about 2 cm below the cotyledons). The inoculation part was wrapped with sterilized soaked absorbent paper. Then wrapped soybean seedlings with tin foil to keep them moist. The experiment was repeated 3 times, 5 biological replicates were set each time. At last, the plants were grown under long-day conditions (16-h light/8-h dark cycles) at 25°C with a light intensity of 170 μmol·m^-2^·s^-1^, and the disease incidence was observed and recorded at different time intervals. Finally, a total of 2 cm hypocotyl was taken from the inoculation site as the material for RNA extraction.

### Identification and sequence analysis of GmRALF family members in soybean

To identify *RALF* genes in soybeans, the RALF1~RALF34 protein sequences from the TAIR database (https://www.arabidopsis.org/) were downloaded as the query sequences (Berardini et al., 2015). Soybean RALF homologs were screened by BLASTp from Phytozome v13 database (https://phytozome-next.jgi.doe.gov/) (Goodstein et al., 2012). The soybean genome and genome annotation files were also downloaded from the Phytozome database. The amino acid composition, molecular weight (MW), isoelectric point (pI), instability index, grand average of hydropathicity (GRAVY) and aliphatic index of the identified GmRALF proteins were assessed using the ExPASy website (http://expasy.org/tools/) (Wilkins et al., 1999; Gasteiger et al., 2003). Moreover, the SignalP website (https://services.healthtech.dtu.dk/) sites used for signal peptide prediction (Nielsen, 2017).

### Phylogenetic analyses and classifications of the GmRALF proteins

The RALF protein sequences of *Arabidopsis* and soybean were aligned using Muscle of MEGA 7.0 with the default parameters. Then, a phylogenetic tree was inferred under the neighbor-joining (NJ) method. The identified GmRALF proteins were further categorized into different subfamilies based on the records of RALF subfamily members in the TAIR database. The resulting tree file was visualized with FigTree v1.4.3. DNAMAN v6.0 was used to display the alignment of amino acid sequences.

### Conserved motif and gene structures analysis

The conserved motif scanning of GmRALF proteins was conducted by MEME v5.4.1 (Bailey et al., 2015). The visualization and the Seq Logos of the MEME-motifs were created by Adobe Illustrator 2021. The structure of these genes was visualized by GSDS v2.0 using the soybean gDNA sequences, the CDS sequences and the phylogenetic tree as templates (http://gsds.gao-lab.org/) (Hu et al., 2015).

### *GmRALFs* chromosomal locations, duplication events and divergence rates analyses

According to the information on the soybean genome available on Phytozome, the chromosomal locations and duplications of *GmRALFs* were mapped and displayed with the MG2C (http://mg2c.iask.in/mg2c_v2.1/) (Chao et al., 2015). We used the virtual machine bio-Linux system to search for duplicated genes and duplicated gene pair nucleotide non-synonymous (Ka) to synonymous (Ks) ratios (Ka/Ks) were calculated with TBtools to analyze the evolutionary relationship.

### *Cis*-regulatory element analysis of *GmRALFs*

For the analysis of *cis*-regulatory elements, the 2 kb region upstream of the transcriptional start site was used to predict the *cis*-elements in promoter regions (http://bioinformatics.psb.ugent.be/webtools/plantcare/html/) (Lescot et al., 2002). The diagram of *cis*-elements of *GmRALFs* was displayed by TBtools (Chen et al., 2020).

### qPCR analyses of *GmRALFs*

The specific quantitative RT-PCR primers for the selected *GmRALFs* were designed by Sangon Biotech (Shanghai, China). The total RNA was extracted by using the RNA prep pure plant kit (Tsingke, Beijing, China) from the frozen samples. All RNA was analyzed by electrophoresis and then quantified with a Nano Vue Plus (Biochrom, Harvard Bioscience Company, UK). The Mighty Script Plus First Strand cDNA Synthesis Master Mix (gDNA digester) (Sangon Biotech, Shanghai, China) was adopted to remove the genomic DNA and convert the total RNA to cDNA. The SYBR qPCR Master Mix (Vazyme Biotech, Nanjing, China) was adopted to conduct the quantitative RT-PCR assay on a QuantStudio™ 6 Flex real-time system (Thermo Fisher Scientific, USA). Triplicate quantitative assays were performed on each cDNA sample. The soybean *GmActin* gene was used as internal control in reactions. The 2^-△△CT^ comparative CT method was used to estimate the relative expression level of genes. Students’ t-test was used for statistical analysis. Information on the qRT-PCR primer sequences can be found in **Supplementary Table 1**.

### Functional verification of secretory protein signal peptide

To verify the secretion function of GmRALFs, yeast YTK12 was used. About 30 amino acids encoding regions from the N-terminal of GmRALFs were cloned into pSUC2 and then transformed into yeast YTK12 by the lithium acetate method (Gietz et al., 1995). Whether the protein contains a signal peptide can be judged by the growth of the strain on CMD-W and YPRAA media and the color reaction of 2, 3, 5-triphenyl tetrazolium chloride (TTC) (Yin et al., 2018). The primers information can be found in **Supplementary Table 1.**

### Effect of GmRALF peptides on disease resistance of soybean

The fragments of encoding sequences of the GmRALF4 peptide (amino acids 26~114) and GmRALF24 (amino acids 26~115) peptide were amplified from the cDNA of Williams 82. All the *GmRALF* genes were amplified using primers with BamH I and Sal I restriction sites at the 5’ and 3’ ends. After digestion, the *GmRALF* genes were inserted into a modified expression vector, *pCold-ProS2*, containing a His-ProS2 tag, before being transformed into the *E. coli* strain BL21 Gold (DE3). Expression and purification of GmRALF proteins were performed as described by Zhang et al. (Zhang et al., 2020). The hypocotyls of soybean seedlings grown in darkness for 3 days were soaked with 1 μM GmRALF protein solution (20 mM MES and 100 mM NaCl were used as buffer). After soaking in darkness for 8 h, the hypocotyls were inoculated with *F. oxysporum*. Then, the plants were grown under long-day conditions (16-h light/8-h dark cycles) at 25°C with a light intensity of μmol·m^-2^·s^-1^. Malondialdehyde (MDA) levels were measured as described by Cakmak and Horst (Cakmak and Horst, 1991). Each treatment contained 15 biological replicates. The experiment was repeated 2 times.

### Yeast two-hybrid assay

The coding sequence of *GmFERed*_(79~1353)_, *GmRALF4*_(79~345)_, *GmRALF5*_(1~522)_, *GmRALF10*_(1~159)_, *GmRALF24*_(79~345)_ and *GmRALF25*_(1~387)_ fragments were obtained by PCR amplification and sequencing. The *GmFERed* PCR product was recombined into the EcoR I/BamH I site of the *pGADT7* (*AD*) vector. *GmRALF4*_(79~345)_, *GmRALF5*_(1~522)_, *GmRALF10*_(1~159)_, *GmRALF24*_(79~345)_ and *GmRALF25*_(1~387)_ PCR products were cloned into the EcoR I/BamH I sites of *pGBKT7* (*BD*) vector.

The plasmids of *AD* (empty vector) or *pGADT7-GmFERed* (*AD-GmFERed*) were introduced into the yeast strain AH109. The plasmids of *BD* (empty vector), *pGBKT7-GmRALF4*_(79~345)_ (*BD-GmRALF4*), *pGBKT7-GmRALF5*_(1~522)_ (*BD-GmRALF5*), *pGBKT7-GmRALF10*_(1~159)_ (*BD-GmRALF10*), *pGBKT7-GmRALF24*_(79~345)_ (*BD-GmRALF24*), or *pGBKT7-GmRALF25*_(1~387)_ (*BD-GmRALF25*) were introduced into AH109 containing *AD* or *AD-GmFERed*. Polyethylene glycol/LiAc-mediated yeast transformation was performed according to the protocol of Yeastmaker Yeast Transformation System 2 (Clontech). The interaction was tested on the SD medium without adenine, histidine, leucine and tryptophan (SD-Ade-His-Leu-Trp). TYPE ONE SERINE/THREONINE PROTEIN PHOSPHATASE 4 (TOPP4) interacts with PIN-FORMED 1 (PIN1) in yeast, where it is used as a positive control (Guo et al., 2015). The primers information can be found in **Supplementary Table 1**.

## Results

### Identification, phylogenetic analyses and classifications of *GmRALF* members in soybean

**Figure 1:**
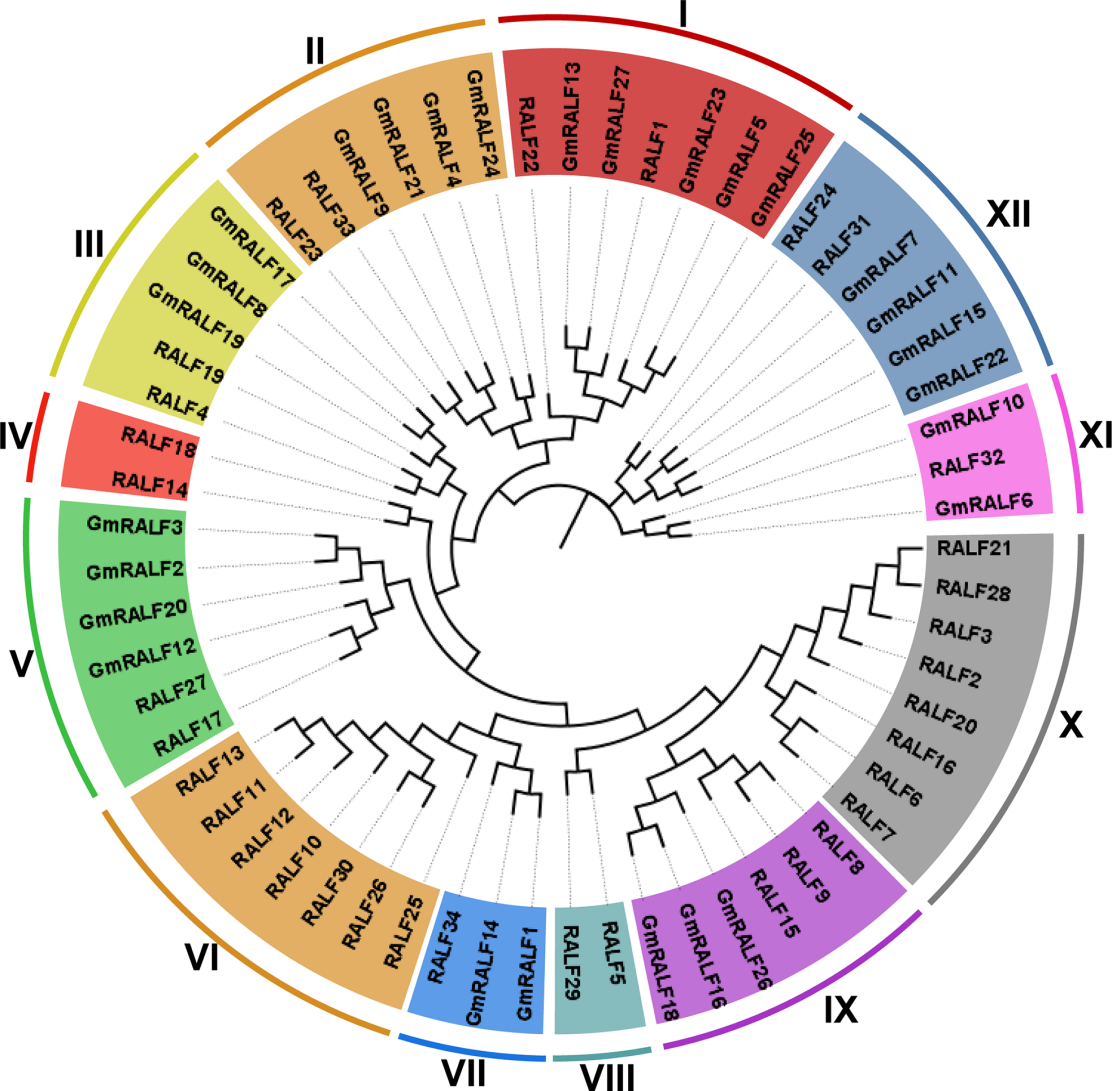
Phylogenetic tree of RALF proteins in soybean and *Arabidopsis*. Full-length protein sequences were aligned using Muscle. The phylogenetic tree was constructed employing the UPGMA method with 1,000 bootstrap values in MEGA 7.0 software, and was optimized with the Figtree v1.4.3. The clades were marked with different colors.

Information from the database showed that the soybean genome contained 27 homologous genes (Wm82.a4.v1). All 27 *GmRALFs* were located on 15 different soybean chromosomes and named *GmRALF1* to *GmRALF27* (**Supplementary Table 2**) according to their chromosomal locations. To classify the phylogenetic relationships of soybean GmRALF proteins, we constructed a phylogenetic tree based on the identified 27 GmRALF proteins and 34 reported Arabidopsis RALF (AtRALF) proteins from TAIR (**Supplementary Table 3**). The phylogenetic tree analysis showed that all AtRALFs were divided into 12 subfamilies (from clade I to clade XII), four of which had no *GmRALFs* (**Figure 1**). Combining the results of amino acid sequence alignment for each clade (**Supplementary Figure 1**), we found that clade I, clade II, clade III and clade VII all contain RXXL (R is arginine, L is leucine and X is any amino acid) cleavage site, YISY, four cysteine residues and RGC(5N)C domains, which were considered necessary for mature RALF. Moreover, RALF1, RALF4, RALF19, RALF23 and RALF34 from the four clades have been shown to participate in cell expansion, pollen tube development and immune response in Arabidopsis, suggesting that *GmRALFs* in the four clades might play a similar role in soybean. The basic characteristics of GmRALF family members were listed in **Supplementary Table 2**, including the protein size, the protein molecular weight (MW), the number of amino acids (aa), the isoelectric point (pI), the instability index, the aliphatic index and the grand average of hydropathicity (GRAVY).

As shown in **Supplementary Table 2**, GmRALF10 is the smallest protein with 53 amino acids (aa), while the largest is GmRALF1 and GmRALF5 with 174 aa. The MW of the proteins ranged from 6.04 kDa (GmRALF10) to 19.73 kDa (GmRALF5), the pI ranged from 6.94 (GmRALF23) to 9.91 (GmRALF10), the aliphatic index ranged from 49.81 (GmRALF10) to 101.35 (GmRALF15) and the instability index spanned from 19.69 (GmRALF8) to 64.03 (GmRALF4). 19 GmRALF proteins had an instability index greater than 40, indicating that the 19 proteins might be unstable. The predicted GRAVY results showed that there were four proteins with GRAVY greater than 0, indicating that all except these four proteins were probably hydrophilic proteins. Additionally, GmRALFs had significantly more amino acids and an average protein molecular weight than RALF. However, there were no significant differences in pI, instability index, the aliphatic index and GRAVY between GmRALFs and their homologs in Arabidopsis, indicating that the physicochemical properties of GmRALFs and RALFs are generally similar. Meanwhile, signal peptides of GmRALF proteins were predicted. The results showed that only three of all the GmRALF proteins are devoid of the signal peptides, indicating that they may not have a secretory function. The coding sequences and the protein sequences of the identified GmRALFs were listed in **Supplementary Table 4**.

### Gene structures and motif patterns of GmRALF members

**Figure 2:**
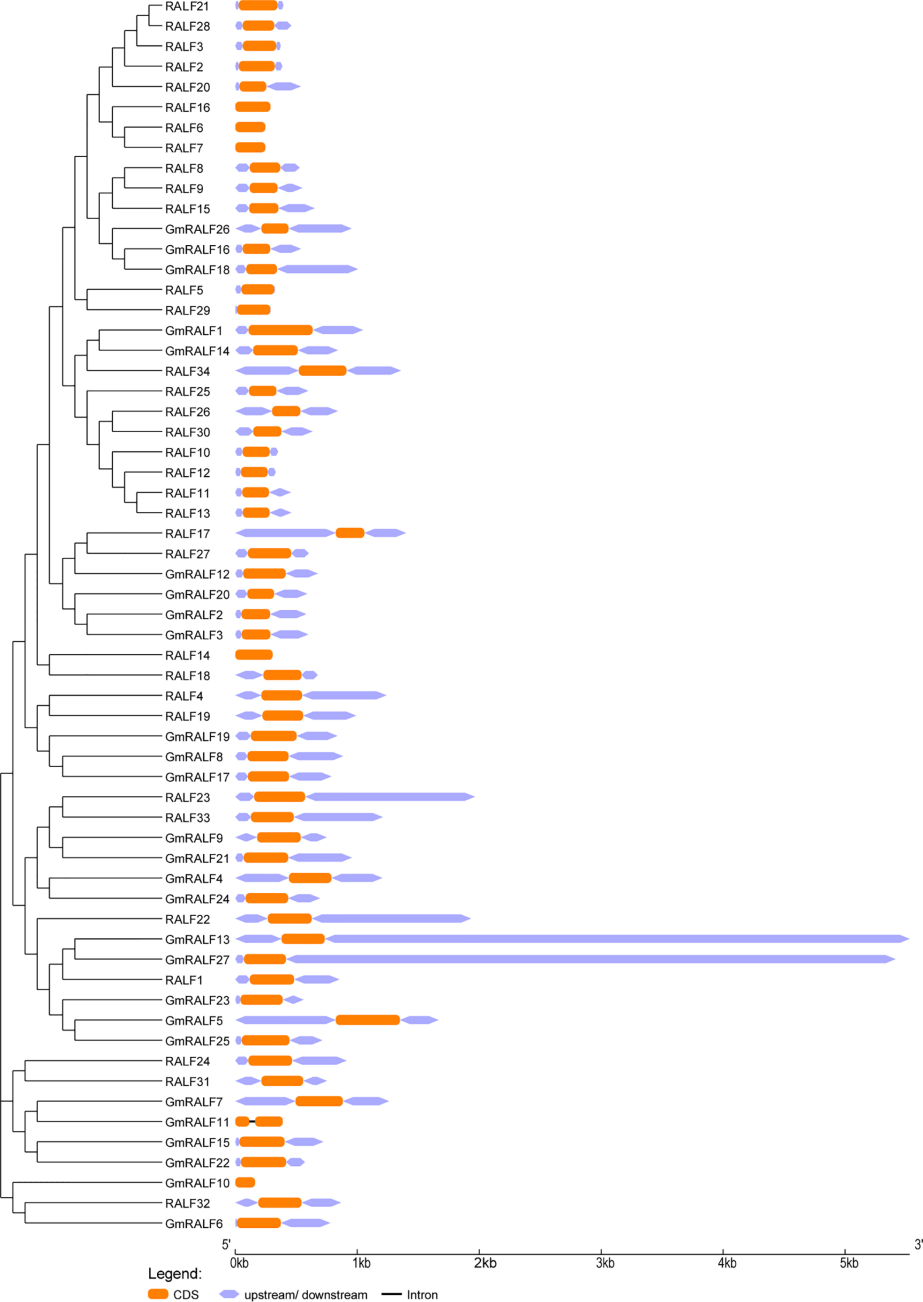
Phylogenetic clustering and gene structures of the *RALF* members in soybean and *Arabidopsis*. Left panel: phylogenetic clustering of the *GmRALF* members. The *GmRALF* members were classified into eight subfamilies. Right panel: gene structures of the *GmRALF* members. Orange boxes indicated the exons; Purplish boxes indicated un-translated 5′- and 3′-regions; black lines indicated introns.

The exon-intron patterns of the identified *RALFs* were obtained by screening the corresponding genomic DNA sequences and annotation files in order to investigate the diversity of *RALF* gene structures. All *RALFs* in soybean and Arabidopsis displayed one to two exons, as seen in **Figure 2**. One intron exists only in *GmRALF11*. The longest gene is *GmRALF13*, and the shortest one is *GmRALF10*. Furthermore, the upstream and downstream coding regions for *GmRALF10*, *GmRALF11*, *RALF6*, *RALF7*, *RALF14* and *RALF16* were absent. The *RALF* gene structure is generally considered to be conservative, with a stable number of exons and introns, but the gene length varied greatly.

**Figure 3:**
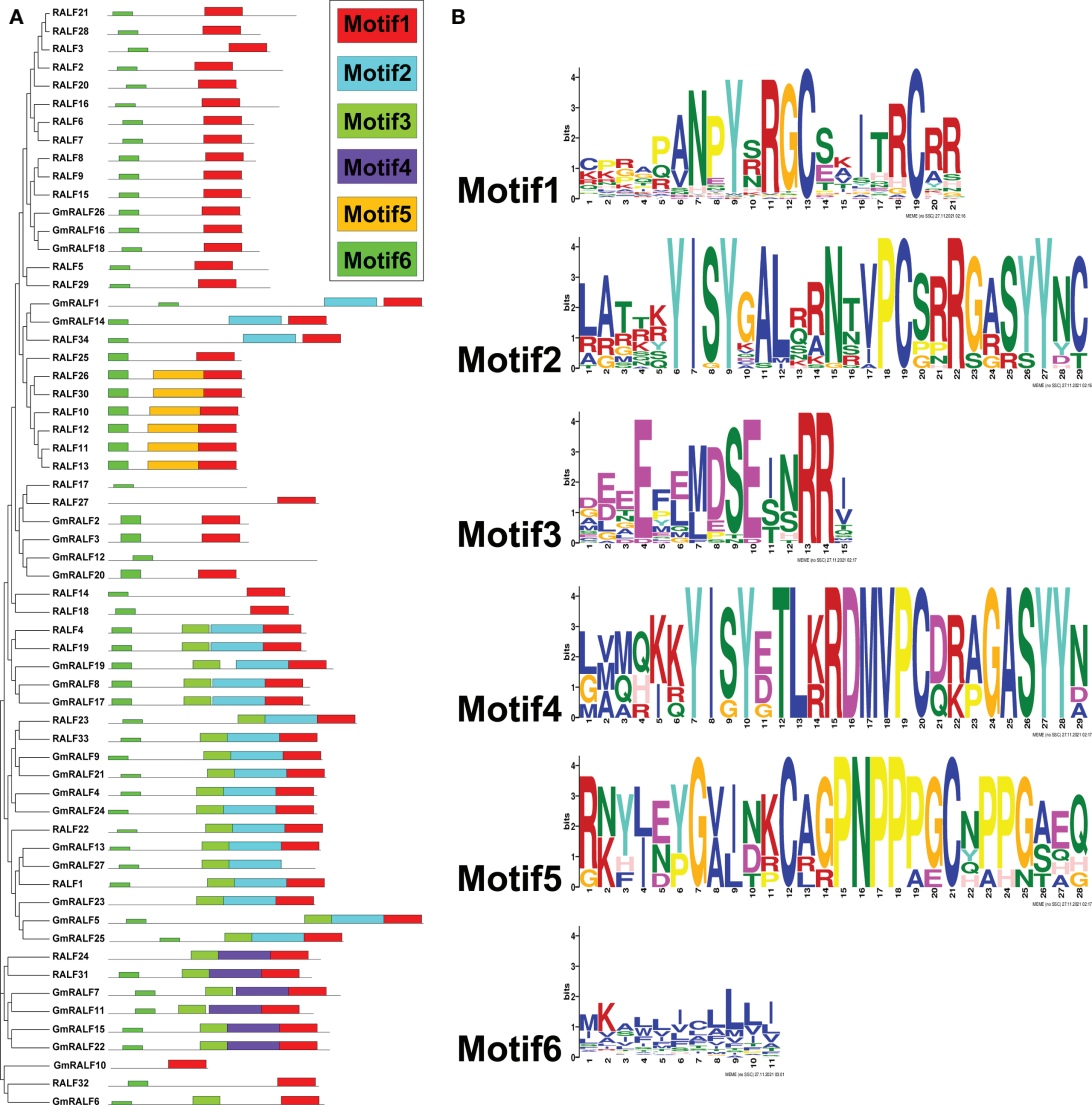
Phylogenetic clustering, the motif patterns and the six motifs of the RALF members in soybean and *Arabidopsis*. **(A)** Phylogenetic clustering and motif patterns of the GmRALF members. The six distinct MEME motifs were displayed in different colored boxes. **(B)** The six motifs in the research.

To further demonstrate the structures of the GmRALF proteins, a scheme was built-up based on the MEME-motif scanning result. As shown in **Figure 3**, six diverse MEME-motifs (named motif1 to motif6) were displayed. The details of these motifs were presented in **Supplementary Table 5**. Nearly all RALFs in soybean and Arabidopsis contain motif1, which is found at the C-terminus of these proteins and contains the RGC(5N)C and RCRR domains. Notably, we discovered that motif3 typically coexists with motif2 or motif4. The RXXL site, which S1P can identify, can be formed by motif3-motif2 or motif3-motif4. After being cut by S1P, RALFs become mature functional proteins. And the YISY and GASYY domains of motif2 and motif4 serve as significantly conserved motifs in mature RALFs. The representative RALF members RALF1, RALF4 and RALF23, which control plant growth and development, have motif3 and motif2. The results suggested that the GmRALFs with both motif3 and motif2 or motif3 and motif4 may need to be cleaved by S1P to form mature GmRALF proteins. Besides, motif5 only exists in a few Arabidopsis RALFs, whose functions have not been reported. The majority of the amino acids in motif6 found at the N-terminus of RALFs are hydrophobic. This finding implies that motif6 may be connected to the formation of signal peptide. This also inspired us to explore whether GmRALFs have a secretory function.

### Chromosomal distributions analyses of *GmRALF* members

**Figure 4:**
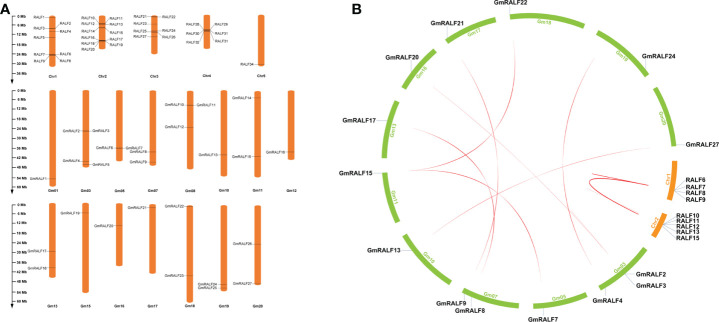
Gene location and synteny. **(A)** The chromosomal distributions of *RALFs* in soybean and *Arabidopsis* genome. The *GmRALFs* were not evenly distributed in 15 soybean chromosomes. The chromosome names were set at the top of the chromosomes. The length of each chromosome can be estimated by using the scale on the left hand. **(B)** Synteny analysis of *RALF* genes between soybean and *Arabidopsis*.

Based on the physical location information of the soybean genome, the chromosome positions of *GmRALFs* were described (**Figure 4A** and **Supplementary Table 6**). Twenty-seven *GmRALFs* are randomly distributed on 15 soybean chromosomes (**Figure 4A**). The soybean chromosomes Gm02, Gm04, Gm06, Gm09 and Gm14 do not have any *GmRALFs* on them. Notably, four genes are located on chromosome Gm03, which harbors the majority of *GmRALFs*. Gm08 contains three *GmRALFs*. Gm05, Gm07, Gm11, Gm13, Gm19 and Gm20 contain two *GmRALFs*, while the others contained only one *GmRALF*. There was no significant correlation between the chromosome length and the number of *GmRALFs*.

Gene duplication plays an important role in increasing the numbers of genes and their subsequent evolution. Hence, to better understand the evolutionary selection of the RALFs family, we analyzed RALFs duplication events using MCSanX software (**Figure 4B** and **Supplementary Table 7**) and calculated Ka/Ks (**Supplementary Table 8**) of the *RALFs* in soybean and *Arabidopsis*. Ultimately, we identified 10 paralogous gene pairs in *Arabidopsis*, and eight orthologous pairs in soybean. These pairs include *RALF6*/*RALF7*, *RALF8*/*RALF9*, *RALF8*/*RALF15*, *RALF9*/*RALF15*, *RALF10*/*RALF11*, *RALF10*/*RALF12*, *RALF10*/*RALF13*, *RALF11*/*RALF12*, *RALF11*/*RALF13*, *RALF12*/*RALF13*, *GmRALF2*/*GmRALF3*, *GmRALF3*/*GmRALF20*, *GmRALF4*/*GmRALF24*, *GmRALF7*/*GmRALF15*, *GmRALF8*/*GmRALF17*, *GmRALF9*/*GmRALF21*, *GmRALF13*/*GmRALF27*, *GmRALF15*/*GmRALF22*. In this study, 14 *GmRALFs* on 11 soybean chromosomes contain 8 tandem repeat events, suggesting that these regions are hotspots for the distribution of *GmRALFs*. Notably, most tandem duplication events happened in clade II, clade V and clade VII. In summary, most *GmRALFs* possibly originated from gene duplications.

In this study, the Ka/Ks (non-synonymous substitution/synonymous substitution) ratios of the *GmRALFs* orthologous gene pairs in soybean and *Arabidopsis* were calculated to evaluate the evolutionary constraints acting on the *GmRALFs*. All *GmRALF* gene pairs displayed Ka/Ks values < 1. Therefore, we speculated that the *GmRALFs* might go through strong purifying selective pressures during their evolution (Xie et al., 2018).

### *Cis*-regulatory elements analyses of soybean *GmRALFs*

**Figure 5:**
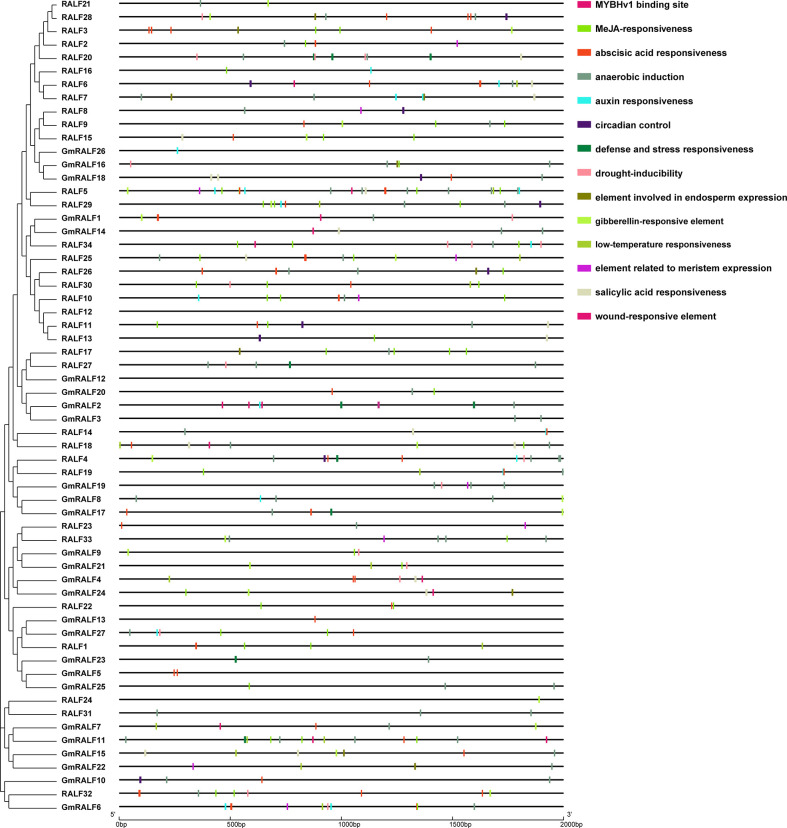
*Cis*-elements in the RALF promoter regions in soybean and *Arabidopsis*. Left panel: phylogenetic clustering of the *GmRALF* members. Right panel: the pattern of the *cis*-elements in the 2 kb upstream hereditary regions of the identified *GmRALFs*. Different *cis*-elements were indicated by distinct colored rectangles.

The *cis*-regulatory elements play an important role in the regulation of gene transcription. In this study, 2 kb upstream sequences of the identified *GmRALFs* were extracted from the soybean genome and analyzed using PlantCARE website for *cis*-elements analysis (**Supplementary Table 9**). A total of 62 different *cis*-elements were obtained in the 2 kb promoter regions of *GmRALFs*. It is important to note that, as depicted in **Figure 5**, the promoter regions of *GmRALFs* have numerous *cis*-elements, including MeJA, defense, abscisic acid, low-temperature, drought and salicylic acid (ABA) responses, suggesting that the *GmRALFs* are differentially induced by various abiotic stresses and control various biological processes. It has been reported that poplar *PdRALF2* can be instantaneously suppressed by MeJA (Haruta and Constabel, 2003). In *Arabidopsis*, *RALF1* also played a key role in regulating the response of FER to ABA signaling. When *RALF1* expression is inhibited, the activation of FER is inhibited, and plants showed high sensitivity to ABA (Chen et al., 2016). It is known that MeJA signaling plays a role in both plant growth and immunity. However, most of these studies were concentrated on how do *RALFs* control plant growth. This prompted us to investigate whether *GmRALF*s, which has numerous stress response components, also regulate the immune response in soybean.

### Expression response of *GmRALFs* to *F. oxysporum* infection

**Figure 6:**
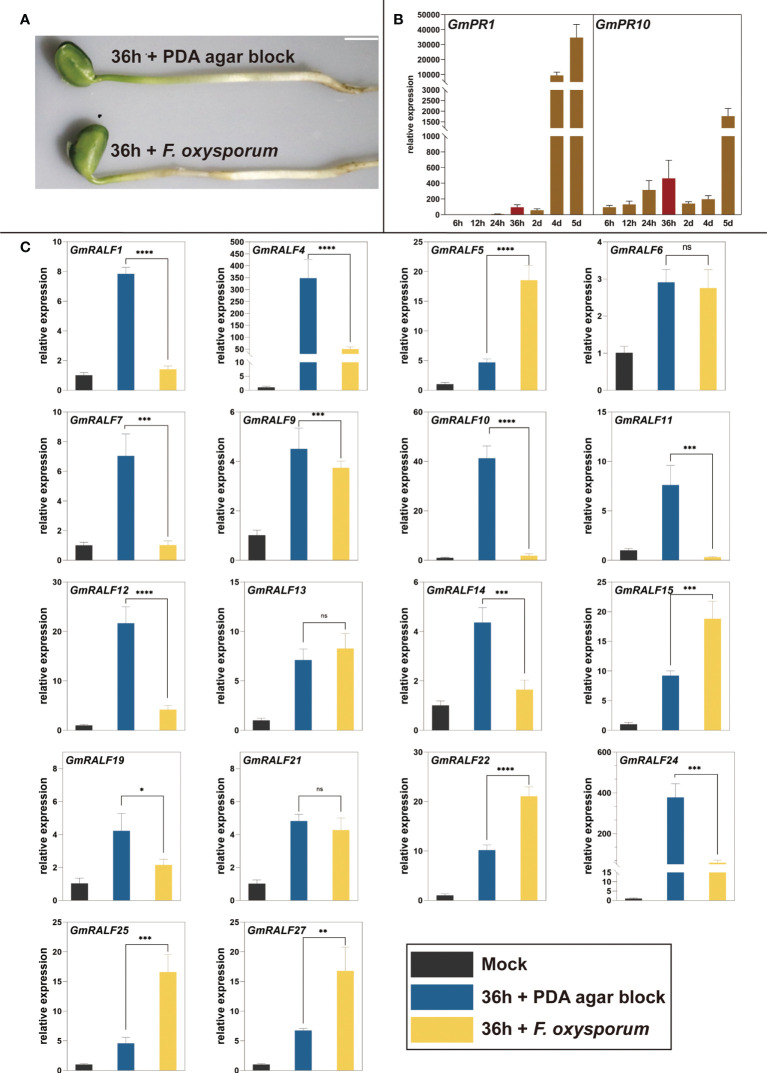
qPCR analysis of *GmRALFs* in response to F. oxysporum infection. **(A)** Disease phenotypes of Jiuyuehuang and control at 36 h after inoculated with F. oxysporum. **(B)** Relative expression level of immune marker genes in soybean treated with F. *oxysporum*. **(C)** Expression patterns of the selected *GmRALFs* after being treated with *F. oxysporum*. *Mock*: uninoculated soybean seedlings at 0 h; *36* h *+ PDA agar block*: soybean seedlings were treated with PDA medium agar block for 36h; *36* h *+* F. *oxysporum*: soybean seedlings were inoculated with F. *oxysporum* for 36 h Data were normalized to the *GmActin* gene, and the experiments all above were repeated three times along with at least three independent repetitions of the biological experiments. Scale bar represents 1 cm. Error bars indicate the standard error. Asterisks indicate statistically significant differences (*P < 0.05, **P < 0.01, ***P < 0.001, ****P< 0.0001, ns, no significance).

Based on the structural and *cis*-elements analysis of *GmRALF* members, we hypothesized that *GmRALFs* might be involved in regulating the soybean immune response. *F. oxysporum* was used to inoculate soybean seedlings. The hypocotyls were more seriously destroyed when inoculated with *F. oxysporum* for 36 h rather than the uninoculated seedlings (**Figure 6A**). Furthermore, the expression of immune response marker genes *GmPR1* and *GmPR10* increased significantly (**Figure 6B**). Therefore, we choose 36 h as the appropriate infection time. Eighteen of all the *GmRALFs* were detectable at the transcription level (**Figure 6C** and **Supplementary Figure 2**). The results showed that the expression was significantly down-regulated in *GmRALF1*, *GmRALF4*, *GmRALF7*, *GmRALF10*, *GmRALF11*, *GmRALF12*, *GmRALF14*, *GmRALF19* and *GmRALF24*. In contrast, the expression levels of *GmRALF5*, *GmRALF15*, *GmRALF22*, *GmRALF25*, and *GmRALF27* were up-regulated. The expression levels of *GmRALF6*, *GmRALF9*, *GmRALF13* and *GmRALF21* were almost not inducible. Notably, four-fold increase in the expression of *GmRALF5* and *GmRALF25*, which are highly homologous to *RALF1*. The expression levels of *GmRALF4* and *GmRALF24*, which were highly homologous with the *RALF23* gene, were down-regulated by about seven-fold. The expression levels of *GmRALF10* were down-regulated about 20 folds. Overall, *GmRALF* family members have different response patterns to *F. oxysporum* infection. The expression patterns of the entire *GmRALF* family indicated that *GmRALF* members might be differentially involved in immunity to *F. oxysporum*.

### Analysis of secretory function of GmRALF proteins

As is known, RALFs are small secreted peptides. Almost all *Arabidopsis* RALF proteins have signal peptides. For instance, after being cut by S1P, the PRORALF23 becomes the mature RALF23 and is then secreted into the extracellular space to regulate the immune response (Stegmann et al., 2017). Five genes, including *GmRALF4*, *GmRALF5*, *GmRALF10*, *GmRALF24* and *GmRALF25*, were chosen to investigate its secretory function based on RT-qPCR analyses.

A yeast mutant strain YTK12 was used to verify the secretory function in this study. The strain lacks sucrose invertase and Trp synthesis genes. Therefore, it could not survive in the CMD-W medium lacking Trp and YPRAA medium with raffinose as the sole nitrogen source. The vector plasmid used in the experiment is *pSUC2* (Jacobs et al., 1997; Oh et al., 2009). *pSUC2* not only contains the Trp synthetic gene but also contains a sucrose transferase lacking signal peptide. Therefore, YTK12 containing *pSUC2* empty plasmid could grow on a CMD-W medium. But it wouldn’t grow in YPRAA. In addition, the color reaction could be used to verify whether proteins have a secretory function, because the invertase enzymatic activity can be detected by the reduction of 2,3,5-Triphenyltetrazolium Chloride (TTC) to insoluble red-colored 1,3,5-Triphenylformazan (TPF) (Yin et al., 2018). If the fusion plasmid containing signal peptide is transferred into yeast strain YTK12, the sucrose invertase gene can be synthesized and secreted into the medium to help its growth. And 0.1%TTC could also be used to stain the fusion plasmid-carrying strains. We used the proven secretory effector Avr1b as a positive control (Shan et al., 2004). The negative control was Mg87, a non-secretory protein from *Magnaporthe grisea* (Gu et al., 2011).

**Figure 7:**
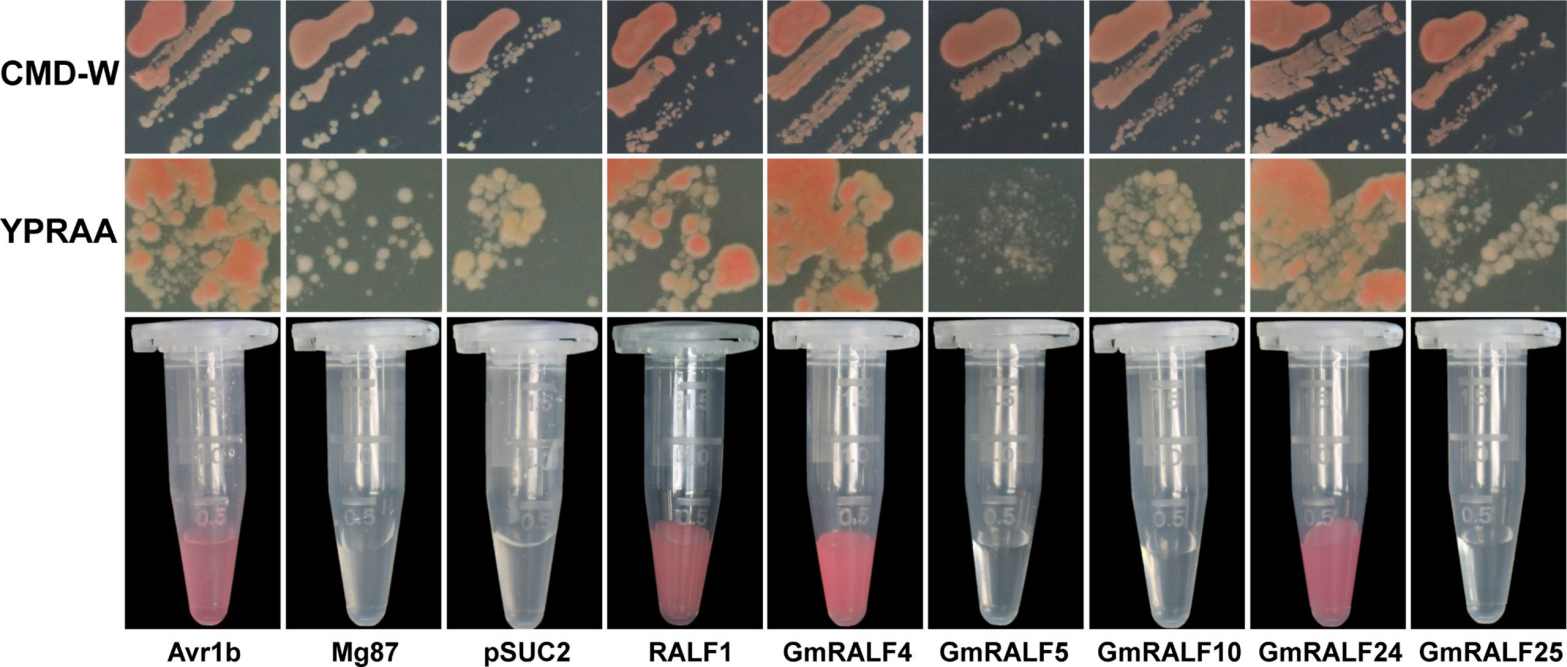
Analysis of the secretory function of selected GmRALF members. The yeast YTK12 strain carrying GmRALF4 and GmRALF24 signal peptides fragment fused in the pSUC2 vector were able to grow in both the CMD-W and YPRAA media, and also induce a red color reaction, indicating the secretory function. Others contained no secretory function. Avr1b was a secreted protein and served as a positive control in this study. Mg87, a *Magnaporthe oryzae* protein, was shown to have no secretory function in 2011 and was here used as a negative control.

Our results showed that (**Figure 7**), the yeast YTK12 strain carrying GmRALF4 or GmRALF24 signal peptides fragment fused in the pSUC2 vector can grow in both the CMD-W and YPRAA media, and can also induce a red color reaction, indicating the secretory function. We also detected RALF1, and turned out that RALF1 contains a signal peptide. Consequently, GmRALF4 and GmRALF24 are secretory peptides, whereas GmRALF5, GmRALF10 and GmRALF25 are not, suggesting that GmRALFs might function in different ways. And this result was consistent with the pre-dictionary results of the signal peptide.

### GmRALF4 and GmRALF24 enhanced the susceptibility of soybean

In order to further explore the effects of GmRALFs on disease resistance in soybean, GmRALF4 and GmRALF24 were expressed in *E. coli* and purified (**Supplementary Figure 3A**). Soybean seedling hypocotyls were immersed in a 1 μM GmRALF4 or GmRALF24 protein solution. After soaking for 8 h, the hypocotyls were inoculated with *F. oxysporum*. The results showed that the soybean seedlings pretreated with GmRALF4 or GmRALF24 had higher susceptibility and higher membrane lipid peroxidation **(** **Supplementary Figures 3B, C** **)**. In summary, GmRALF4 and GmRALF24 increased soybean susceptibility.

### GmRALFs interacts with the extracellular domain of GmFER

RALFs usually regulate plant signaling with their receptor FER in Arabidopsis (Haruta et al., 2014). We wonder whether the FER homolog in soybean also interacts with GmRALFs. We BLAST the soybean homologs in the Phytozome database using the amino acid sequence of AtFER. The results showed that both Glyma.18G215800 and Glyma.09G273300, members of *Catharanthus roseus* RECEPTOR-LIKE KINASE 1-LIKE in *Glycine max* (Gm*Cr*RLK1L) (Wang et al., 2021), are the closest homologues of AtFER. However, only the Glyma.18G215800 was successfully cloned in different tissues of soybean seedlings under various stress conditions. We speculate that Glyma.09G273300 might not be expressed or have other unknown spatiotemporal expression features. Domain analysis showed that Glyma.18G215800 consists of two extracellular malectin domains, a transmembrane domain and an intracellular kinase domain, which is similar to AtFER (**Supplementary Figure 4A**). Due to its similar structure to AtFER, Glyma.18G215800 was named after GmFERONIA (GmFER).

**Figure 8:**
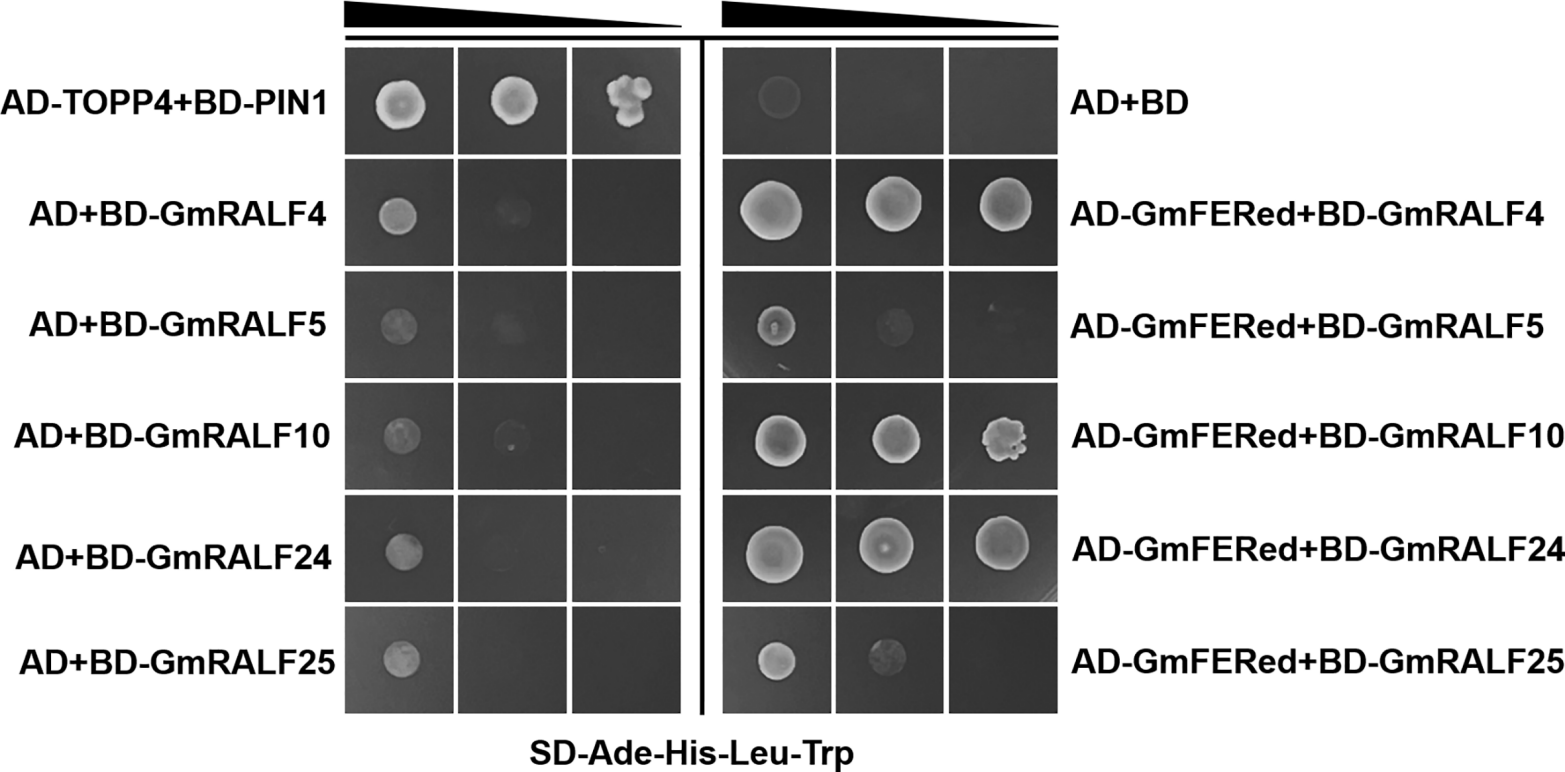
GmRALFs interacts with GmFERed in yeast. GmFERed interacts with GmRALF4, GmRALF10, or GmRALF24. GmRALF4 and GmRALF24 were removed from the signal peptide. The interaction between GmFERed and GmRALFs was tested on synthetic defined medium without adenine, histidine, leucine, and tryptophan (SD-Ade-His-Leu-His). Serial dilutions of the yeast colonies were plated. The experiment was repeated three times with similar results.

The interaction between GmFER and GmRALF4, GmRALF5, GmRALF10, GmRALF24 or GmRALF25 was predicted using the String online tool (https://cn.string-db.org/). The results showed that the full-length GmFER interacts with GmRALF4, GmRALF5, GmRALF24 and GmRALF25 (**Supplementary Figure 4B**). The yeast two-hybrid results showed that (**Figure 8**) GmRALF4, GmRALF10 and GmRALF24 interact with GmFERed. In contrast, the interaction between GmFERed and GmRALF5 or GmRALF25 was not identified. Importantly, these results are not exactly the same as predicted. However, the distinctive conserved domains of RALF proteins are present in GmRALF5 and GmRALF25. Whether these two GmRALFs interact with other sites of GmFER remains to be explored.

## Discussion

RALFs are a type of protein that are widely involved in plant growth, development and immunity. With the rapid development of whole-genome sequencing technologies, RALF members have been gradually identified in the genomes of species except for plants, such as fungi and nematodes (Masachis et al., 2016; Thynne et al., 2017; Zhang et al., 2020). RALFs were initially found to inhibit root growth as their physiological roles, in addition to alkalization and MITOGEN-ACTIVATED PROTEIN KINASE (MAPK) activation (Pearce et al., 2001b; Haruta and Constabel, 2003).

In this study, 27 *GmRALFs* were identified in soybean (**Figure 4A**) and classified into eight subfamilies according to their structure (**Figure 1** and **Supplementary Figure 1**). *RALF1*, *RALF4* and *RALF23* are the most deeply and widely studied in the *RALF* gene family, which are the representatives in regulating plant growth, development and immunity. *RALF1*, *RALF4* and *RALF23* were divided into clade I, clade III and clade II, respectively. The number of *GmRALFs* in these three clades occupied about half of all the *GmRALFs*. In addition, *GmRALFs* belonging to these three clades all possess the necessary conserved domains including RXXL, YISY, four cysteine residues and RGC(5N)C. Previous studies have identified the function of *RALF1* in inhibiting cell expansion. Overexpression of *RALF1* resulted in plant dwarfism, smaller leaves and shorter roots. The *RALF1* knockout mutant, however, showed the exact opposite phenotype (Bergonci et al., 2014). And RALF23 can inhibit the production of ROS induced by flg22 and destroy the complex structure of BAK1/FLS2. After recognizing flg22, FLS2 interacts with its co-receptor BAK1 to regulate plant immune responses (Chinchilla et al., 2007; Ma et al., 2016; Wang et al., 2020). It is the most direct evidence that *RALFs* can regulates plant immune response (Stegmann et al., 2017; Wang et al., 2020). Moreover, *RALF4* specifically regulates plant reproductive growth such as pollen tube development (Mecchia et al., 2017). We hypothesized that *GmRALF5*, *GmRALF13*, *GmRALF23*, *GmRALF25* and *GmRALF27* might be involved in regulating root growth. *GmRALF4*, *GmRALF9*, *GmRALF21* and *GmRALF24* may be involved in plant immune response besides regulating plant elongation. According to the online analysis of eplants (http://bar.utoronto.ca/eplant_camelina/) (**Supplementary File 1**), excepted for no information *GmRALF21* and *GmRALF23* was identified in the database, all of the aforementioned GmRALF members have a significant expression in the root. With their high similarity to *RALF4* and highly significant expression, *GmRALF8*, *GmRALF17* and *GmRALF19* may play a role in soybean flowers.

Based on the analysis results of replication events, most tandem duplication events happened in clades II, V and VII. Eight tandem duplication events involving nearly 14 *GmRALFs* spread across 11 soybean chromosomes, indicating that these chromosomes may be the hotspots of the *GmRALFs* distribution. The Ka/Ks values for all *GmRALF* gene pairs are less than one. In a word, most *GmRALFs* may be resulted from gene duplications, and the *GmRALFs* may have experienced strong purification pressures during evolution.

In this exploration, we also analyzed the detected *cis*-elements in the promoter region of the *GmRALFs*. As shown in **Figure 5**, *cis*-elements associated with plant stress responses are widespread. Therefore, the *cis*-elements analysis provided clues to explore the functions, especially the genes related to response to different stresses and plant immune responses. Besides, RALF members were reported to be extensively involved in regulating plant development and stress responses (Pearce et al., 2001b; Covey et al., 2010; Haruta et al., 2014; Masachis et al., 2016; Stegmann et al., 2017; Wood et al., 2020). For example, *RALF23* and *RALF34* are involved in regulating plant immune responses (Stegmann et al., 2017). Additionally, RALF-like proteins in fungi were found in regulating plant immune responses (Masachis et al., 2016; Thynne et al., 2017; Wood et al., 2020).

Soybean root rot caused by *F. oxysporum* severely impacts soybean production. To breed high-yielding, disease-resistant soybean varieties is the most efficient and environmentally friendly way to against soybean disease comparing to those traditional techniques. It is necessary to explore the role of *RALF* in soybean. The main pathogen *F. oxysporum* responsible for soybean root rot was chosen to investigate the function of *GmRALFs* in soybean. We examined the relative expression of *GmRALFs* in soybean treated with *F. oxysporum.* The results from qRT-PCR showed that GmRALFs had different response patterns to the *F. oxysporum*. The expression levels of 5 *GmRALFs* were significantly upregulated, while 9 *GmRALFs* were significantly down-regulated. So far, few reports demonstrated that RALFs positively regulates plant immune response. We discovered that five *GmRALFs* that are able to be induced by *F. oxysporum* have an I(7N)E(3N)DSE domain (**Supplementary Figure 5A**). In contrast, this domain hardly exists in the reduced expression of *GmRALFs* (**Supplementary Figure 5B**). Notably, GmRALF4 and GmRALF24 also possess I(7N)E(3N)DSE domain, indicating that there are additional factors may influence the role of GmRALFs in immune responses (**Supplementary Figure 5C**). We also discovered that GmRALF4 and GmRALF24, whose expression are negatively regulated by *F. oxysporum*, have signal peptides while GmRALF5 and GmRALF23 do not (**Figure 7**). Moreover, GmRALF4 and GmRALF24 could increase the susceptibility of soybean to *F. oxysporum*. Hence, we hypothesized that the role of RALF proteins in the immune response is related to their specific domains and secretory functions. Moreover, GmRALF4, GmRALF10 and GmRALF24 can interact with the extracellular domain of GmFER in yeast two-hybrid assay. Surprisingly, despite having significantly fewer N-terminal amino acids than other GmRALFs, GmRALF10 is also able to interact with GmFERed. The interaction between different structural GmRALFs and different sites of GmFER will be one of the interests in our future work.

## Conclusion

In this study, we identified a total of 27 *GmRALFs* in soybean and analyzed their evolutionary relationships. These genes were divided into eight clades, and the individuals within each clade shared structural and motif similarities. Eight tandem duplication events were identified in the *GmRALFs*, suggesting that tandem duplication events might be the primary force in the evolution of the *GmRALFs*. The *cis*-element analysis of the *GmRALFs* family revealed that there are many immune response elements in the *GmRALFs*. Additionally, when soybean was infected by *F. oxysporum*, the relative expression level of *GmRALFs* changed significantly, indicating that *GmRALFs* are involved in soybean defense to fungal diseases. Further, GmRALF4 and GmRALF24 were confirmed to have signal peptides. And they can inhibit the resistance of soybean to *F. oxysporum*. Moreover, these two GmRALFs as well as GmRALF10 interact with GmFER in yeast. They may regulate the immune response of soybean through the GmFER pathway.

## Data availability statement

The original contributions presented in the study are included in the article/**Supplementary Material**. Further inquiries can be directed to the corresponding author.

## Author contributions

JS, HY, XS, and JD were responsible for the conceived and designed the experiments. JD was responsible for designed the experiments and revised the manuscript. YL was responsible for the data analysis, literature search, and manuscript preparation. YL and YC performed the experiments and data analysis. HJ, ZS, and YZ contributed to reagents, materials and analysis tools. All authors read and approved the final version of the manuscript.

## Funding

The work was supported by funding from the National Natural Science Foundation of China (31871552, 32171939) and the Sichuan Innovation Team Project of the National Modern Agricultural Industry Technology System, China (sccxtd-2020-20).

## Conflict of interest

The authors declare that the research was conducted in the absence of any commercial or financial relationships that could be construed as a potential conflict of interest.

## Publisher’s note

All claims expressed in this article are solely those of the authors and do not necessarily represent those of their affiliated organizations, or those of the publisher, the editors and the reviewers. Any product that may be evaluated in this article, or claim that may be made by its manufacturer, is not guaranteed or endorsed by the publisher.

## Supplementary material

Supplementary Figure 1 Amino acid sequence alignment analysis of GmRALFs and RALFs. The same amino acid residues are shown on a blue background. And conserved domains were labeled, including the RXXL site, the YISY domain, the four cysteine residues domain, and the RGC(5N)C domain. The result was visualized by DNAMAN v6.0.

Supplementary Figure 2 Expression profile cluster analysis of *GmRALFs*. Heatmap showing relative expression levels of *GmRALFs* in response to *F. oxysporum* infection. The result was visualized by TBtools.

Supplementary Figure 3 GmRALF4 and GmRALF24 enhanced the susceptibility of soybean. **(A)** The purified GmRALF4-His and GmRALF24-His peptides were detected by Western blot. **(B)** Soybean seedlings were co-infected with GmRALFs and *F. oxysporum*. **(C)** Levels of MAD. *Mock* means hypocotyls were co-infected with the buffer solution and *F. oxysporum*. Error bars indicate the standard error. one-way ANOVA, asterisks indicate statistically significant differences (*P < 0.05, **P < 0.01, ***P < 0.001, ****P< 0.0001).

Supplementary Figure 4 Domain composition and interaction prediction map of GmFER. **(A)** Domain composition of FER in *Arabidopsis* and soybean. **(B)** Prediction of the interaction between GmFER and GmRALFs.

Supplementary Figure 5 Amino acid sequence alignment analysis of GmRALFs involved in regulating immune response. **(A)** Amino acid sequences of five GmRALFs that positively regulate the immune response. **(B)** Amino acid sequences of nine GmRALFs that negatively regulate the immune response. **(C)** GmRALF4 and GmRALF24 were compared with the amino acid sequences of five GmRALFs which positively regulate the immune response.
