# Supplementary figures and images for: Genome-wide characterization of soybean RALF genes and their expression responses to Fusarium oxysporum
Source: Front Plant Sci. 2022 Oct 6;13:1006028. doi: 10.3389/fpls.2022.1006028 (PMC9583537; doi:10.3389/fpls.2022.1006028)

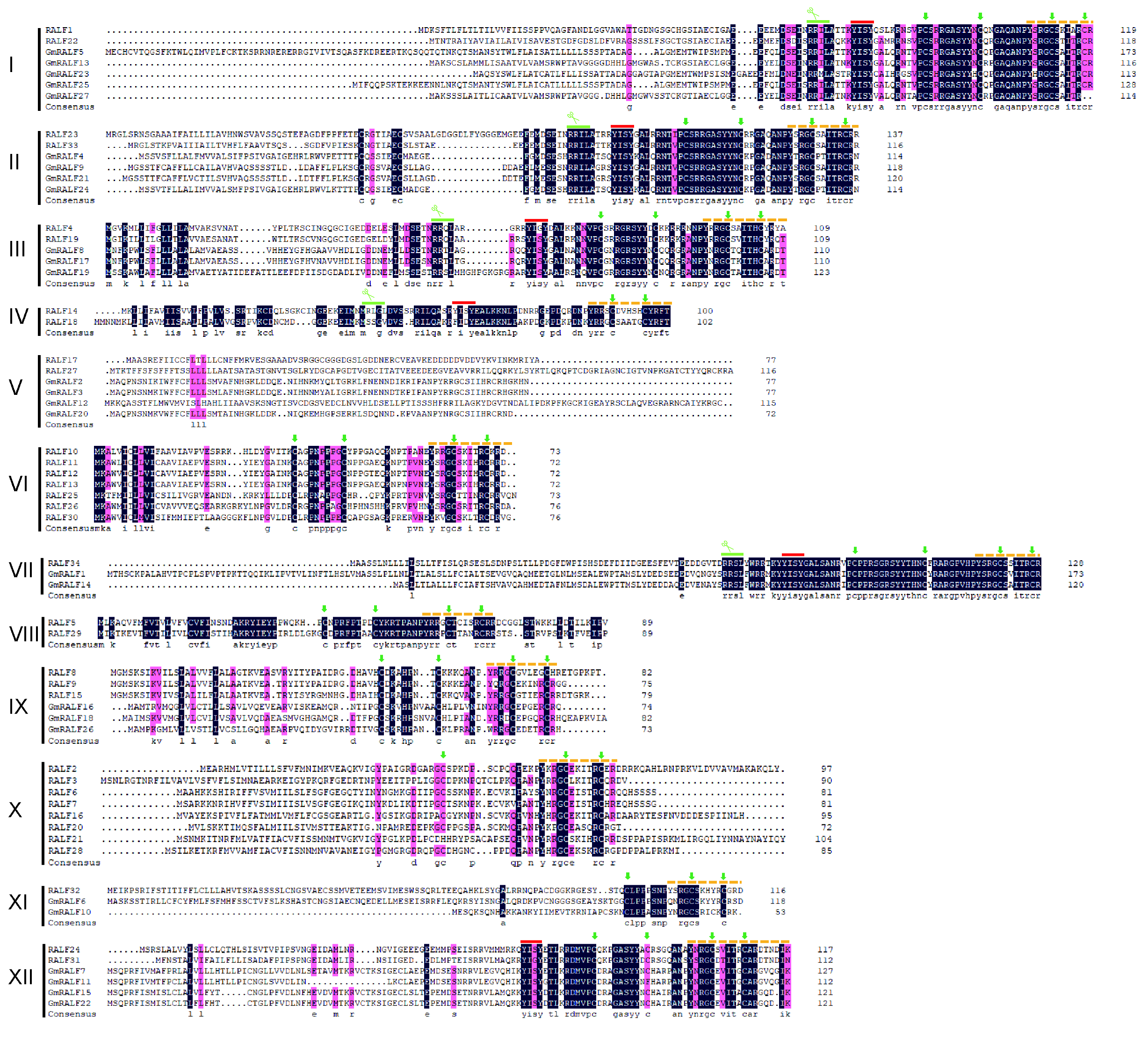

Supplement: Supplementary Figure 1 — Amino acid sequence alignment analysis of GmRALFs and RALFs. The same amino acid residues are shown on a blue background. And conserved domains were labeled, including the RXXL site, the YISY domain, the four cysteine residues domain, and the RGC(5N)C domain. The result was visualized by DNAMAN v6.0. [file Image_1.tif]

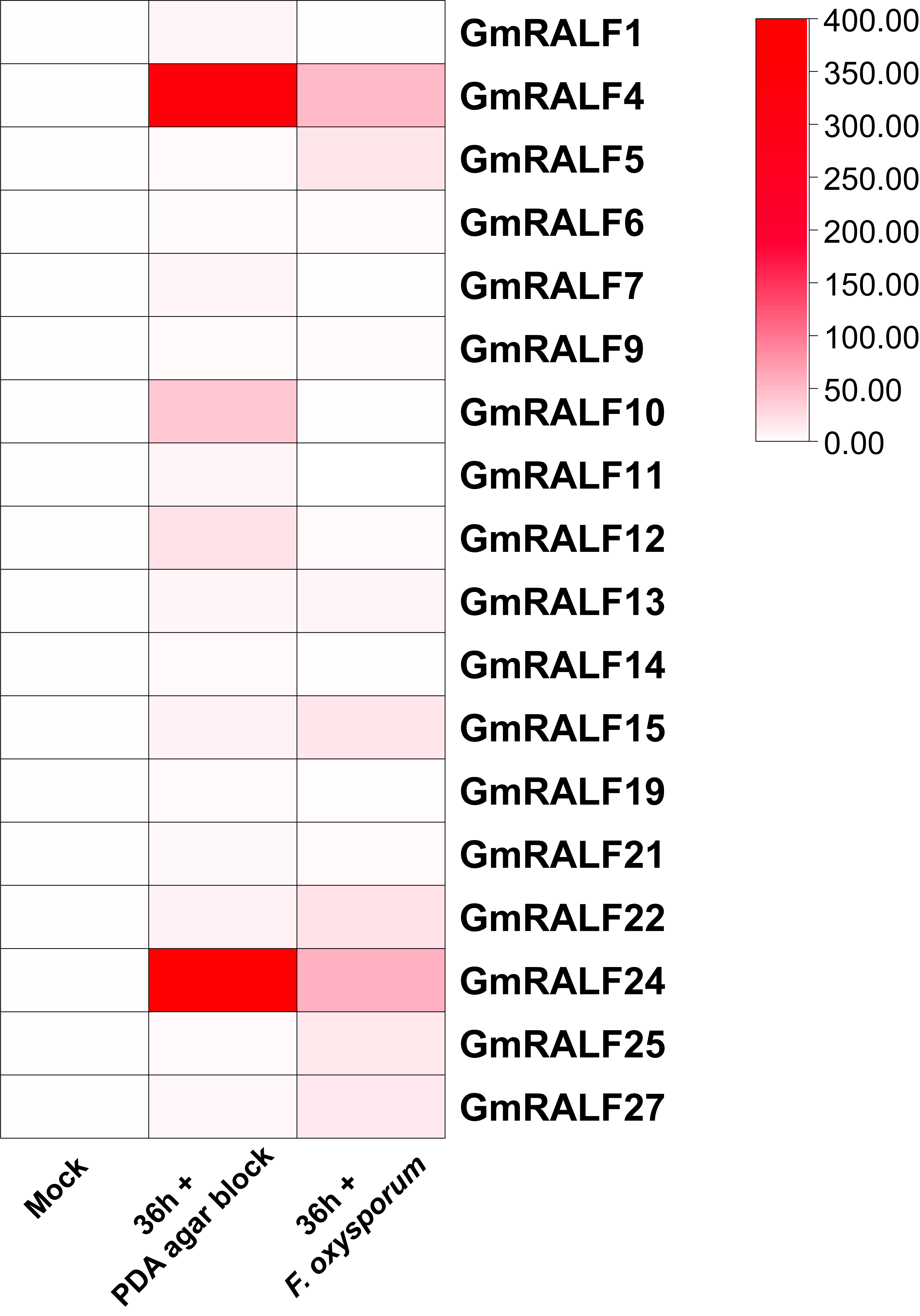

Supplement: Supplementary Figure 2 — Expression profile cluster analysis of GmRALFs. Heatmap showing relative expression levels of GmRALFs in response to F. oxysporum infection. The result was visualized by TBtools. [file Image_2.tif]

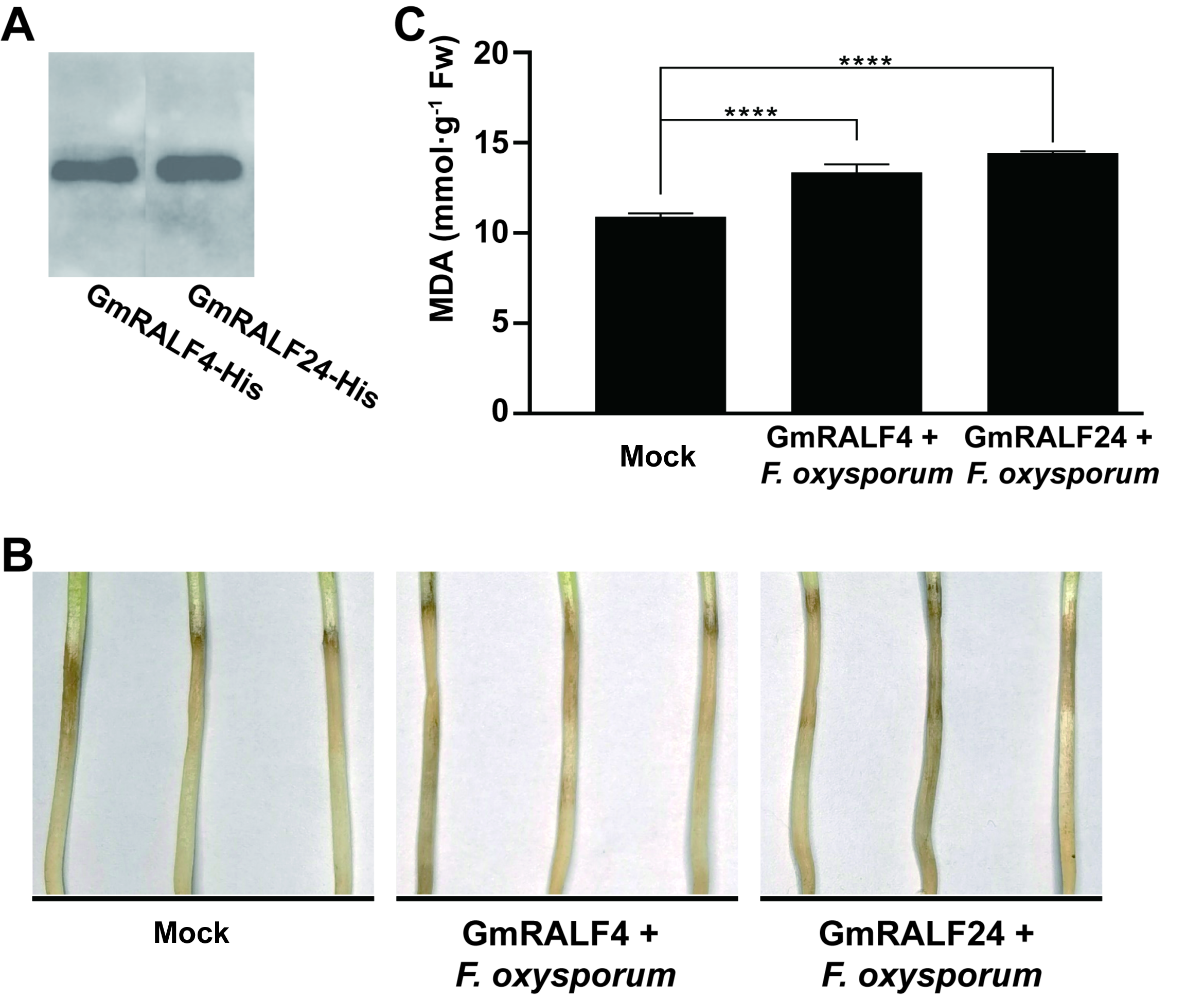

Supplement: Supplementary Figure 3 — GmRALF4 and GmRALF24 enhanced the susceptibility of soybean. (A) The purified GmRALF4-His and GmRALF24-His peptides were detected by Western blot. (B) Soybean seedlings were co-infected with GmRALFs and F. oxysporum. (C) Levels of MAD. Mock means hypocotyls were co-infected with the buffer solution and F. oxysporum. Error bars indicate the standard error. one-way ANOVA, asterisks indicate statistically significant differences (*P < 0.05, **P < 0.01, ***P < 0.001, ****P< 0.0001). [file Image_3.tif]

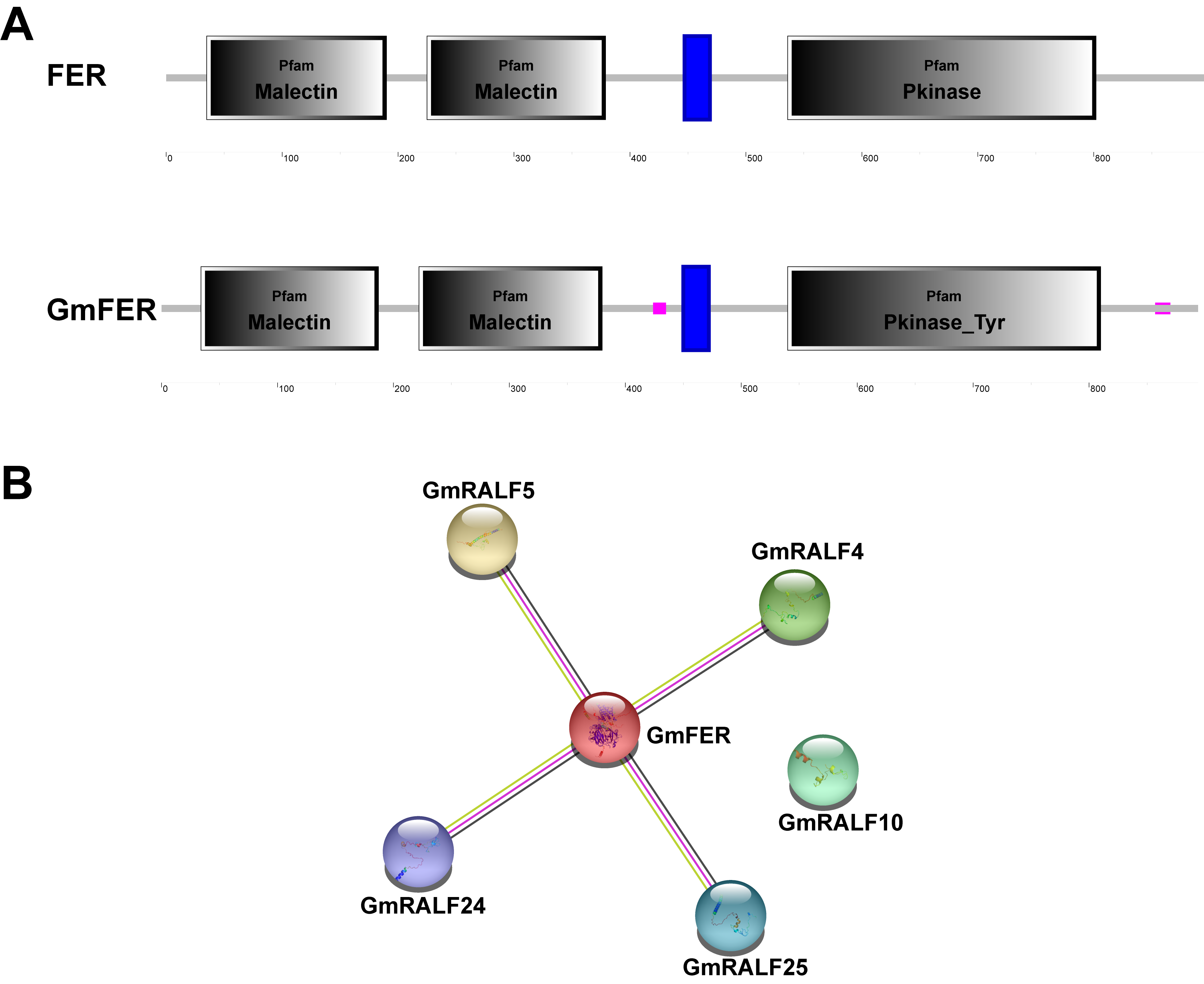

Supplement: Supplementary Figure 4 — Domain composition and interaction prediction map of GmFER. (A) Domain composition of FER in Arabidopsis and soybean. (B) Prediction of the interaction between GmFER and GmRALFs. [file Image_4.tif]

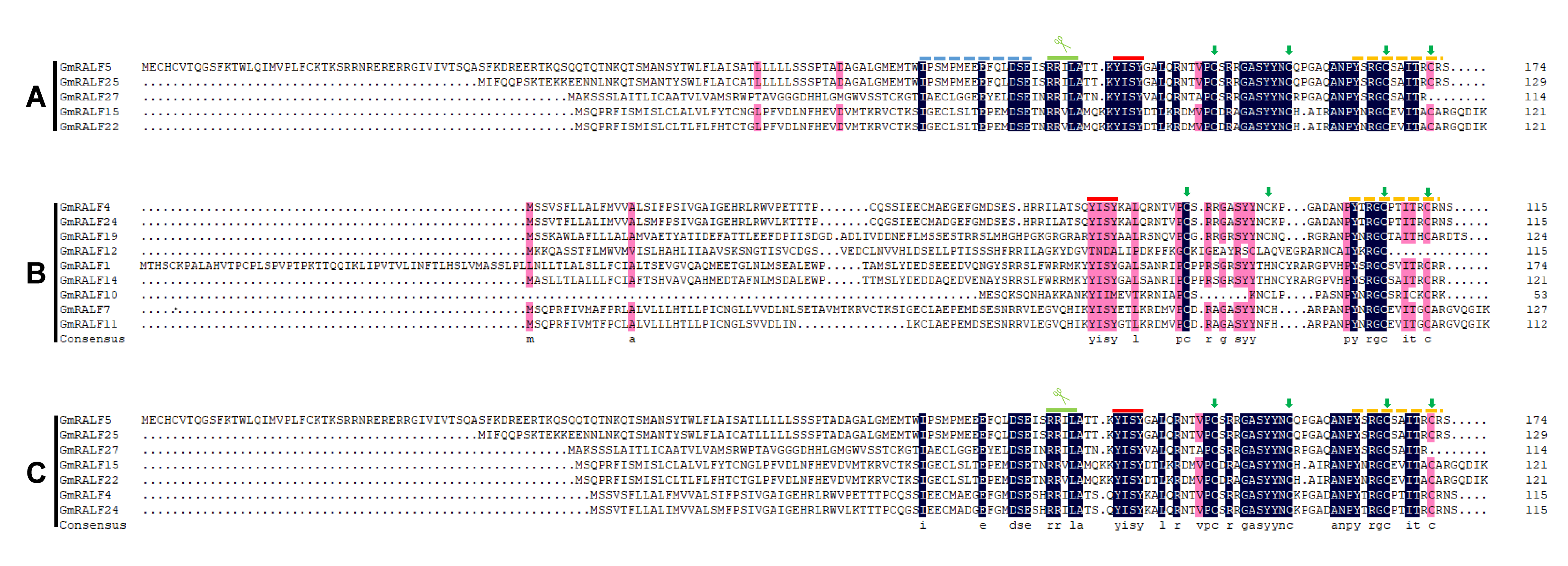

Supplement: Supplementary Figure 5 — Amino acid sequence alignment analysis of GmRALFs involved in regulating immune response. (A) Amino acid sequences of five GmRALFs that positively regulate the immune response. (B) Amino acid sequences of nine GmRALFs that negatively regulate the immune response. (C) GmRALF4 and GmRALF24 were compared with the amino acid sequences of five GmRALFs which positively regulate the immune response. [file Image_5.tif]
