# Supplementary material for: Genome-wide characterization of soybean RALF genes and their expression responses to Fusarium oxysporum
Source: Front Plant Sci. 2022 Oct 6;13:1006028. doi: 10.3389/fpls.2022.1006028 (PMC9583537; doi:10.3389/fpls.2022.1006028)
Supplement: Supplementary file 15 [file DataSheet_1.pdf]

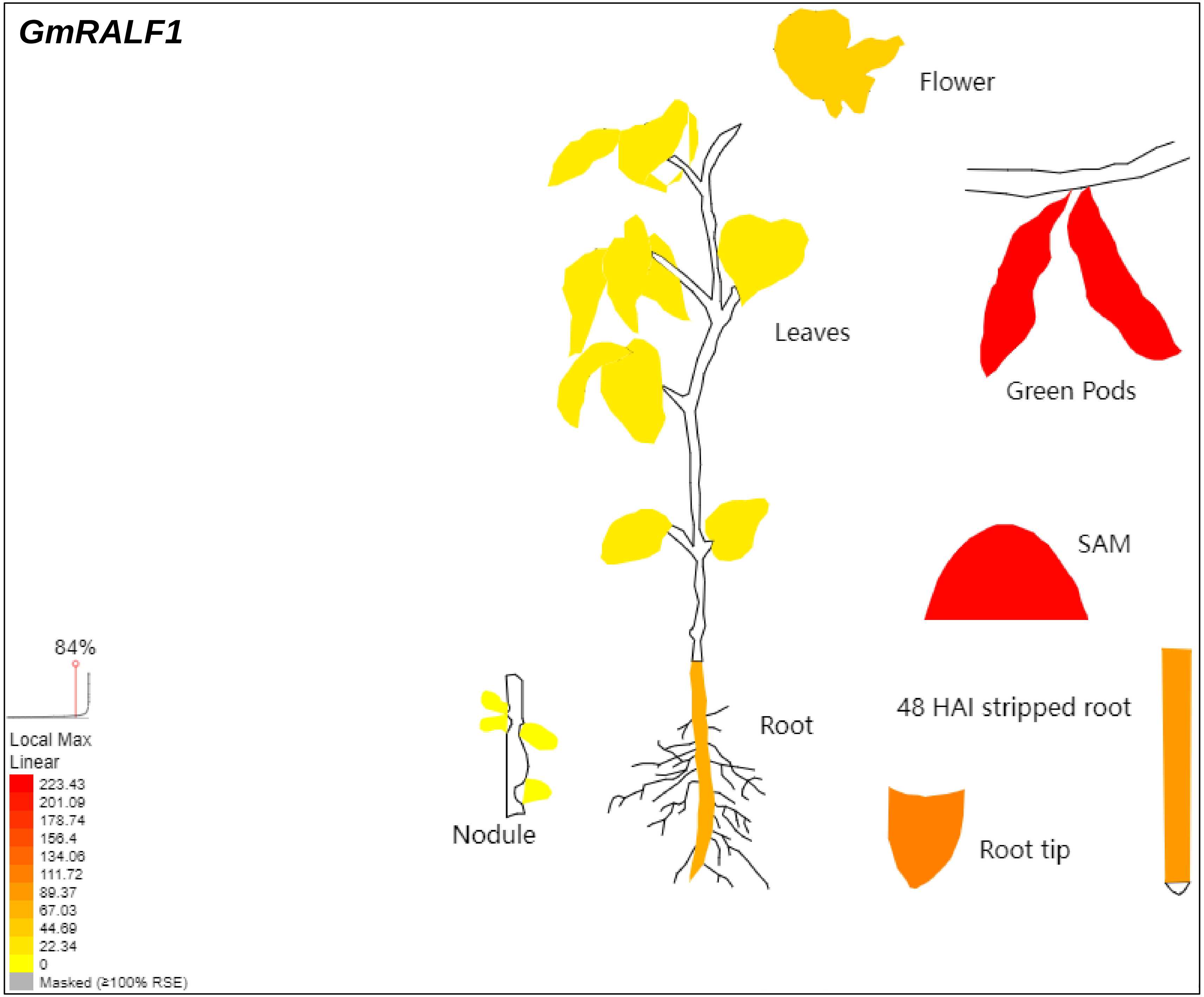
GmRALF1
Flower
Leaves
Green Pods
SAM
84%
Local Max
Linear
223.43
201.09
178.74
156.4
134.06
111.72
89.37
67.03
44.69
22.34
0
Masked (≥100% RSE)
Nodule
Root
48 HAI stripped root
Root tip

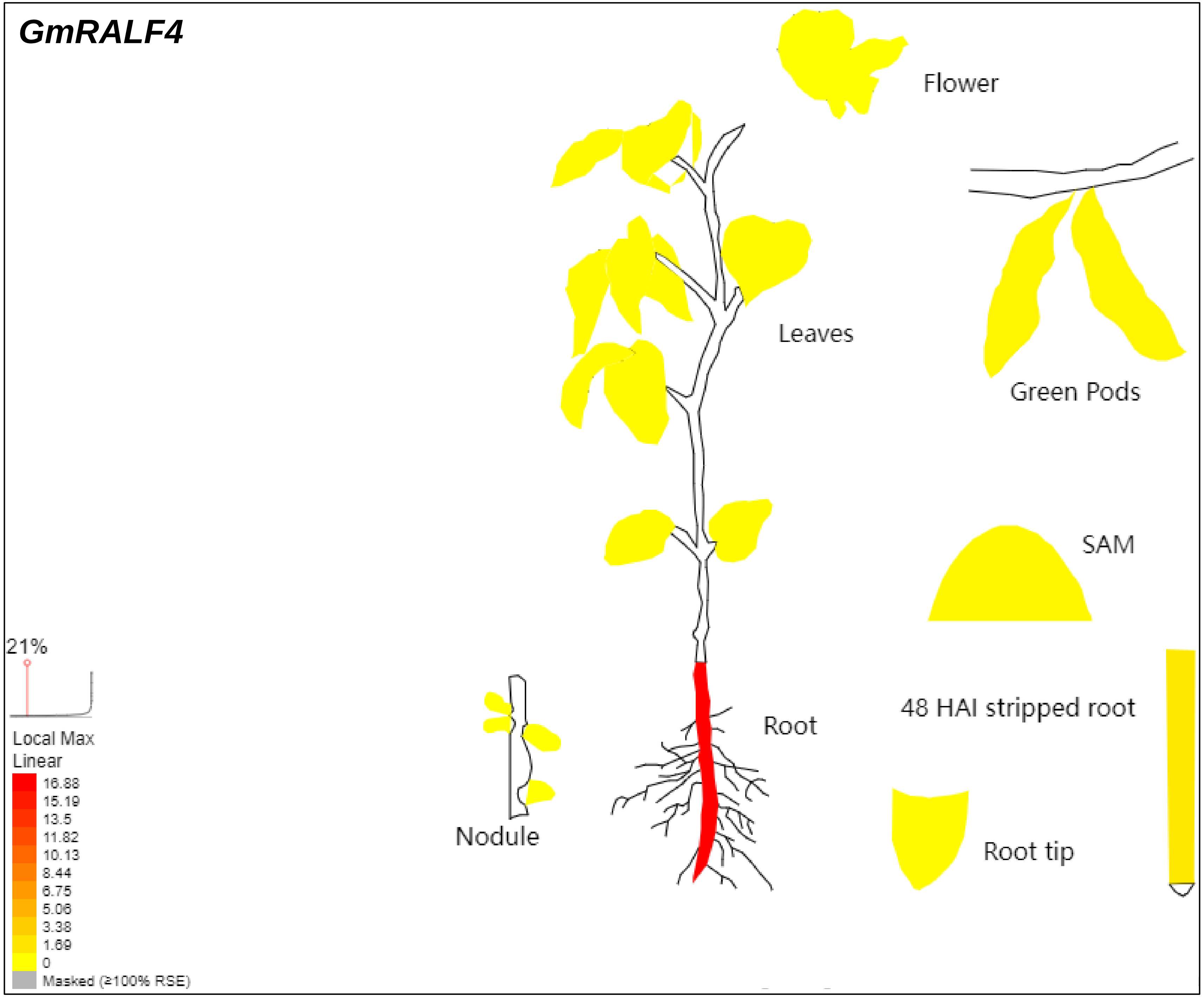

GmRALF4
Flower
Leaves
Green Pods
SAM
21%
Local Max
Linear
16.88
15.19
13.5
11.82
10.13
8.44
6.75
5.06
3.38
1.69
0
Masked (≥100% RSE)
Nodule
Root
48 HAI stripped root
Root tip

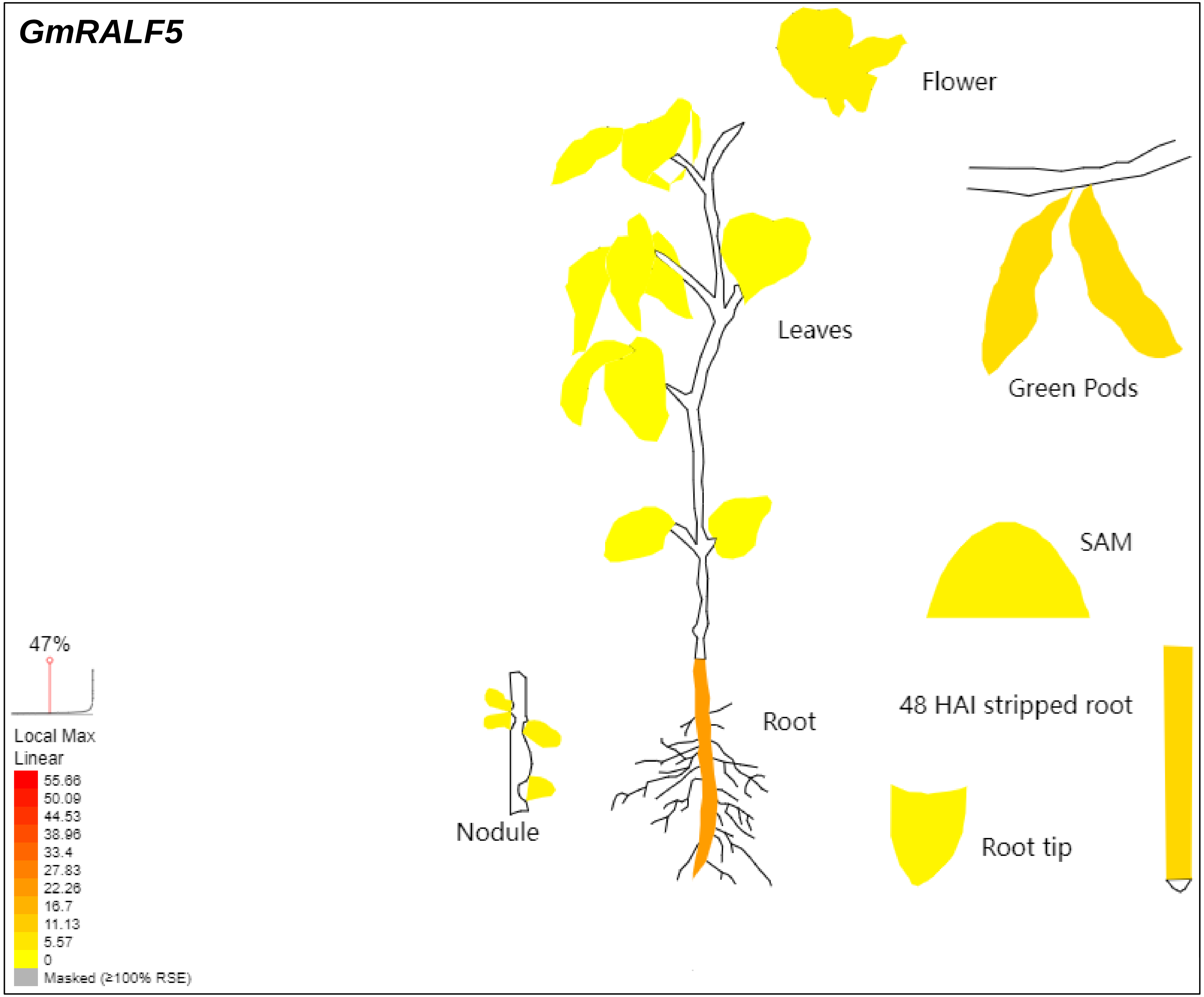
GmRALF5
Flower
Leaves
Green Pods
SAM
48 HAI stripped root
Root
Nodule
Root tip
47%
Local Max
Linear
55.66
50.09
44.53
38.96
33.4
27.83
22.26
16.7
11.13
5.57
0
Masked (≥100% RSE)

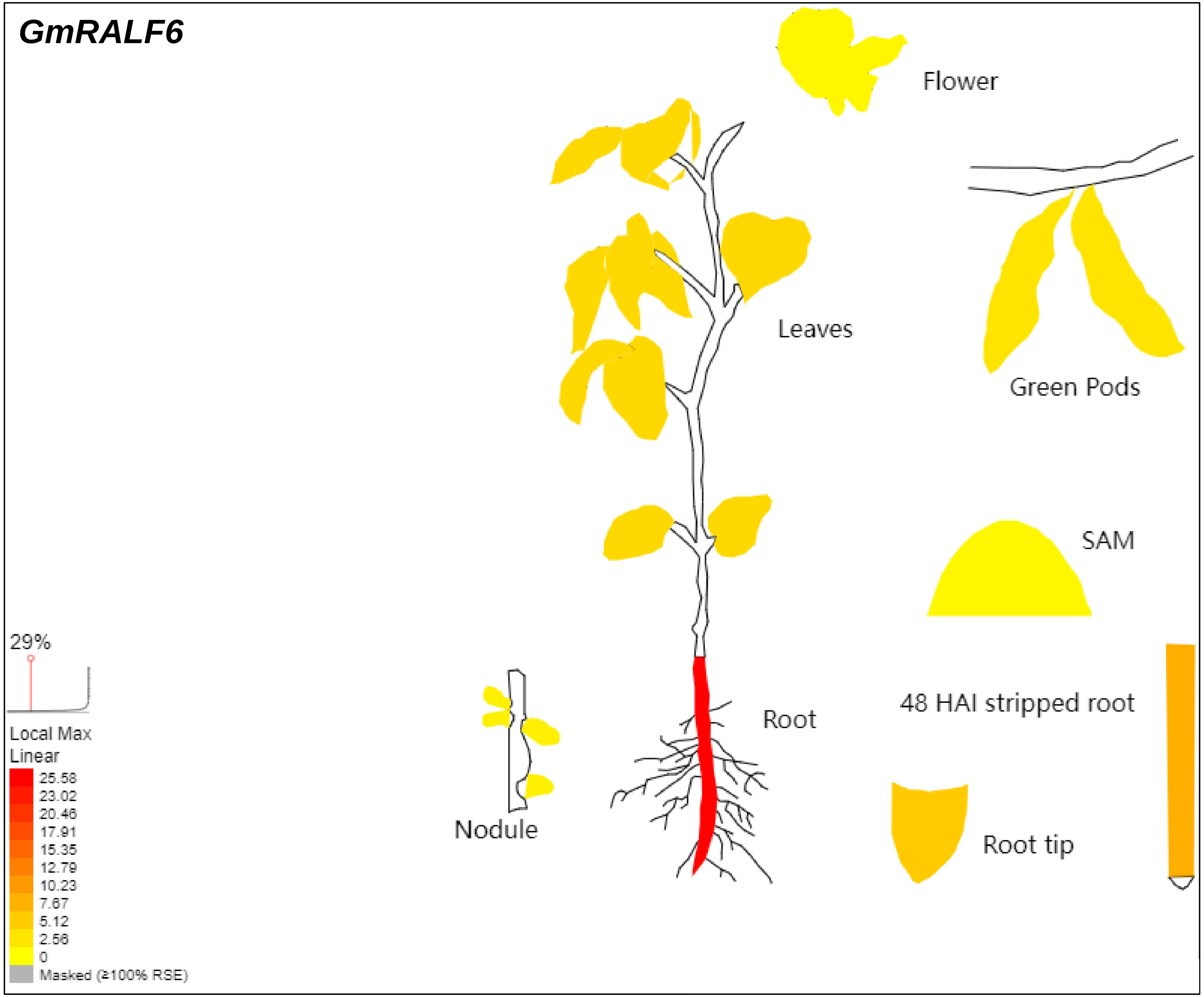
GmRALF6
Flower
Leaves
Green Pods
SAM
Root
48 HAI stripped root
Nodule
Root tip
29%
Local Max
Linear
25.58
23.02
20.46
17.91
15.35
12.79
10.23
7.67
5.12
2.56
0
Masked (≥100% RSE)

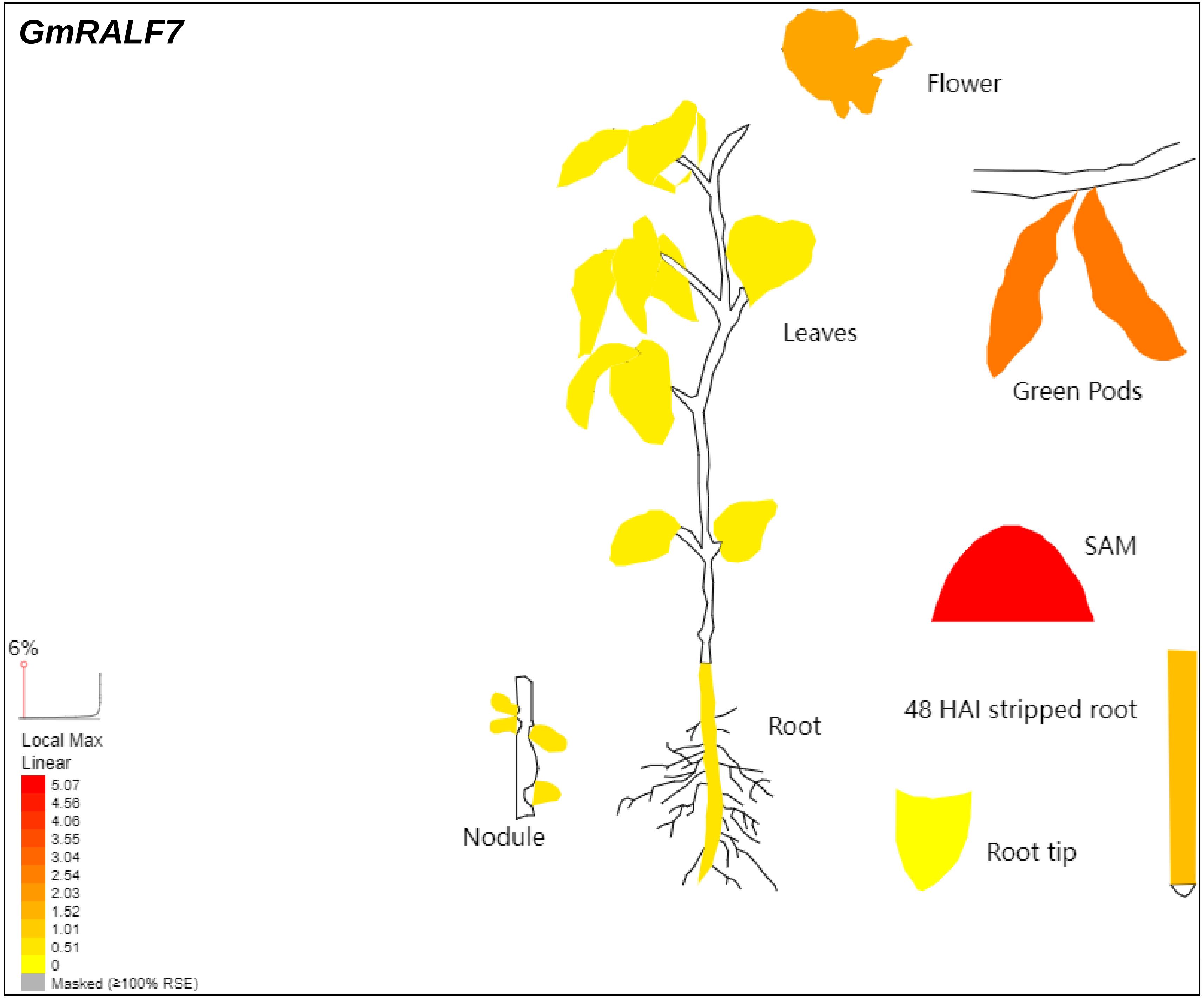
GmRALF7
Flower
Leaves
Green Pods
SAM
48 HAI stripped root
Root
Nodule
Root tip
6%
Local Max
Linear
5.07
4.56
4.06
3.55
3.04
2.54
2.03
1.52
1.01
0.51
0
Masked (≥100% RSE)

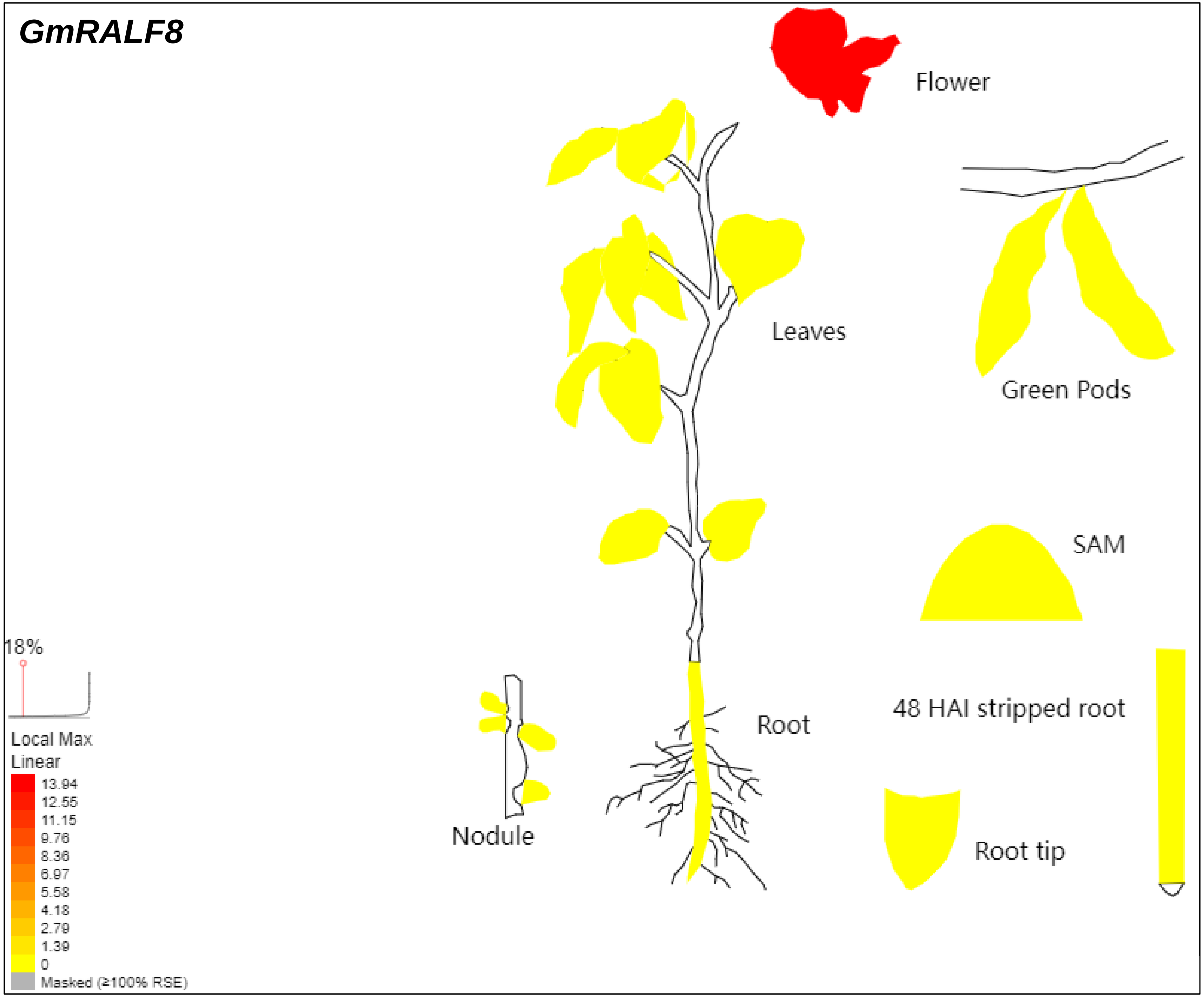
GmRALF8
Flower
Leaves
Green Pods
SAM
Root
48 HAI stripped root
Nodule
Root tip
18%
Local Max
Linear
13.94
12.55
11.15
9.76
8.36
6.97
5.58
4.18
2.79
1.39
0
Masked (≥100% RSE)

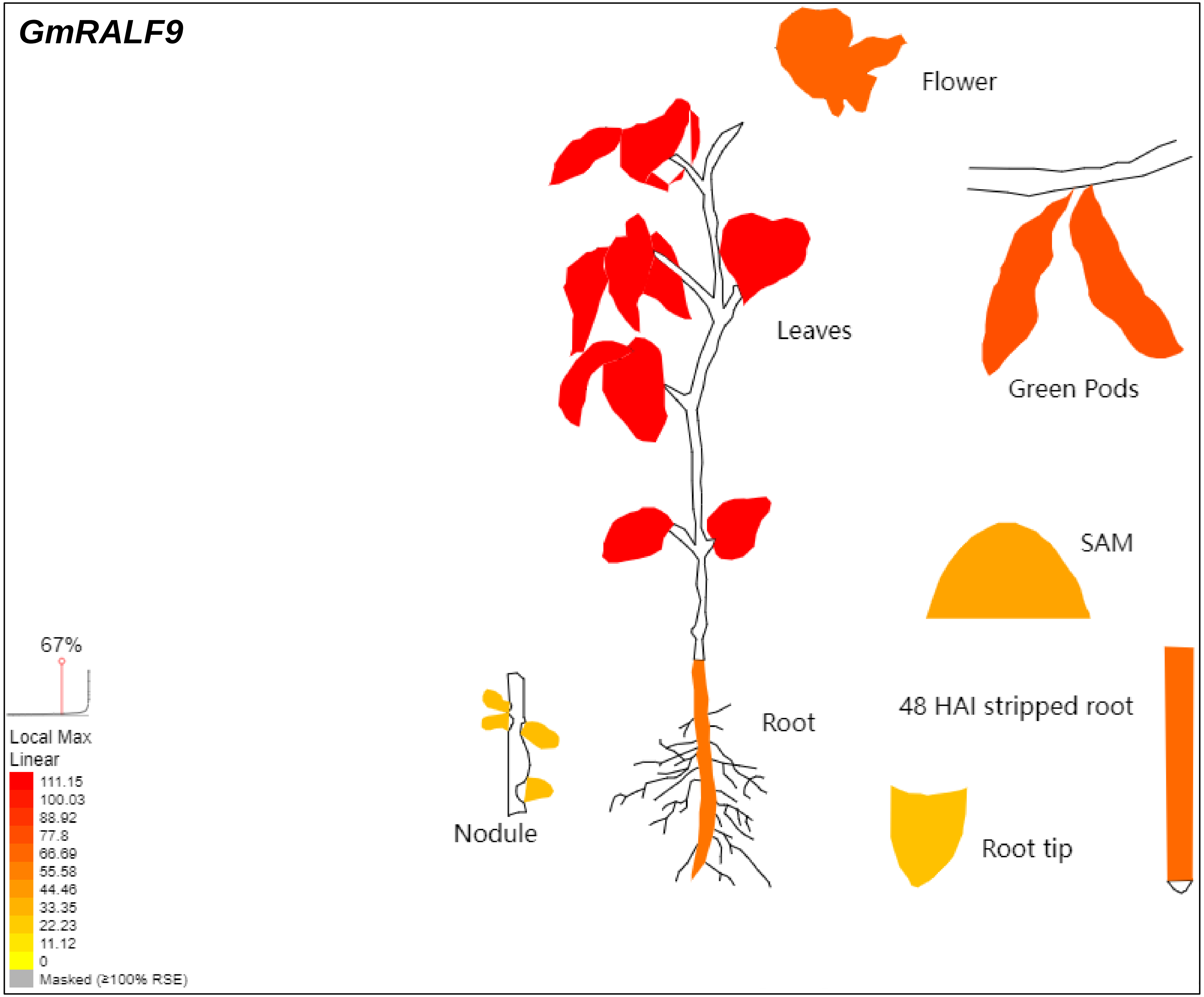
GmRALF9
Flower
Leaves
Green Pods
SAM
48 HAI stripped root
Root
Nodule
Root tip
67%
Local Max
Linear
111.15
100.03
88.92
77.8
66.69
55.58
44.46
33.35
22.23
11.12
0
Masked (≥100% RSE)

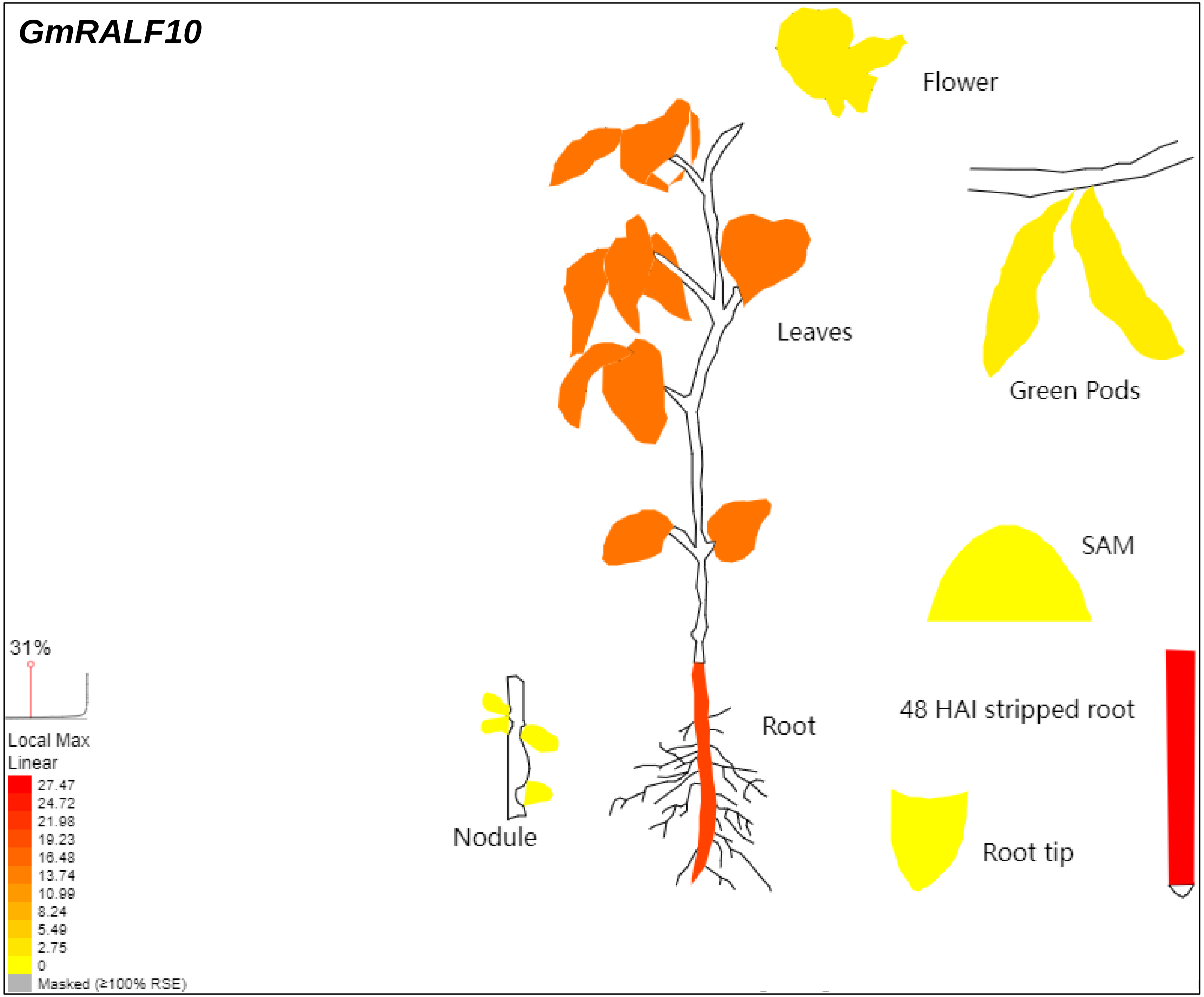
GmRALF10
Flower
Leaves
Green Pods
SAM
48 HAI stripped root
Root
Nodule
Root tip
31%
Local Max
Linear
27.47
24.72
21.98
19.23
16.48
13.74
10.99
8.24
5.49
2.75
0
Masked (≥100% RSE)

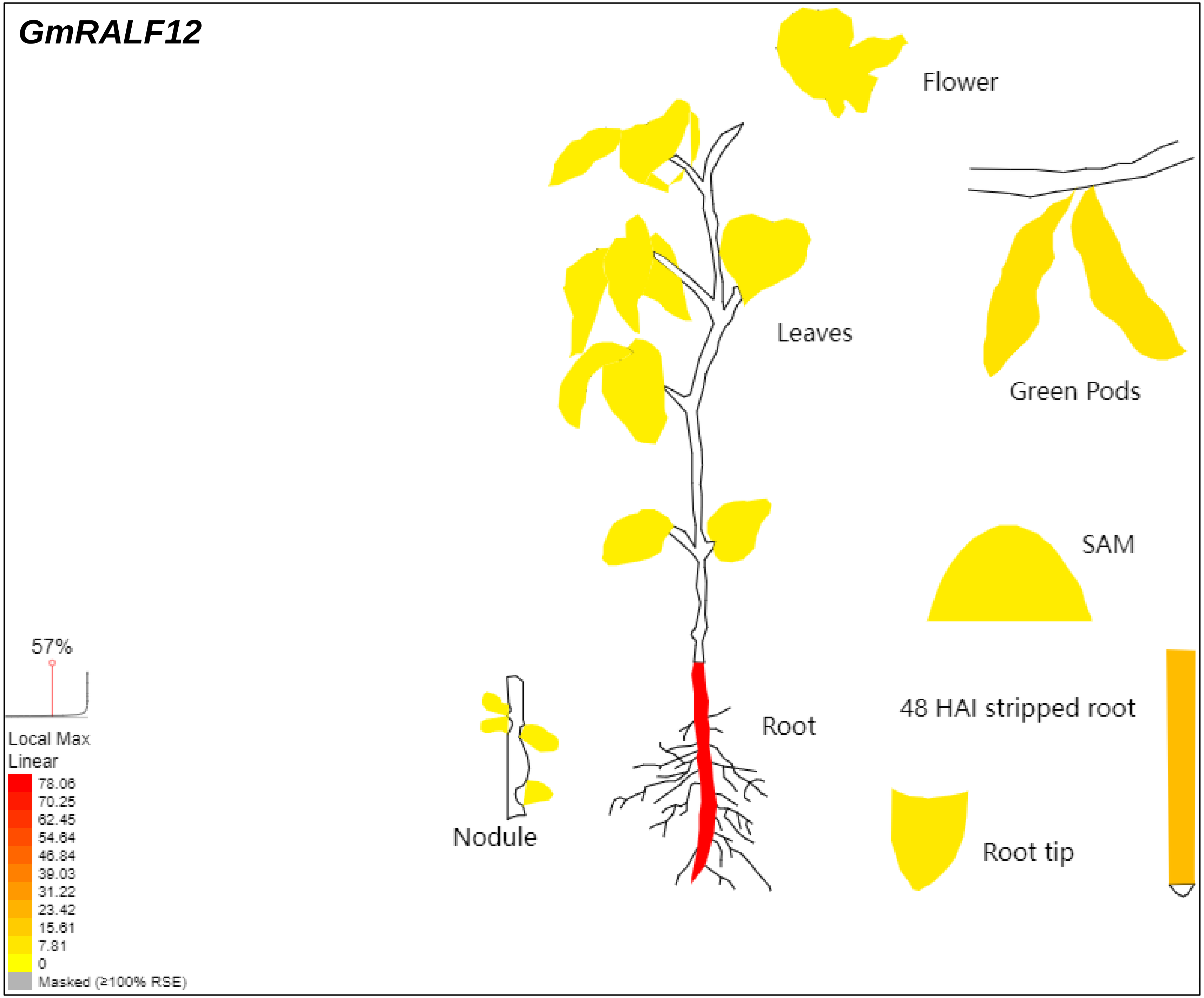
GmRALF12
Flower
Leaves
Green Pods
SAM
48 HAI stripped root
Root
Nodule
Root tip
57%
Local Max
Linear
78.06
70.25
62.45
54.64
46.84
39.03
31.22
23.42
15.61
7.81
0
Masked (≥100% RSE)

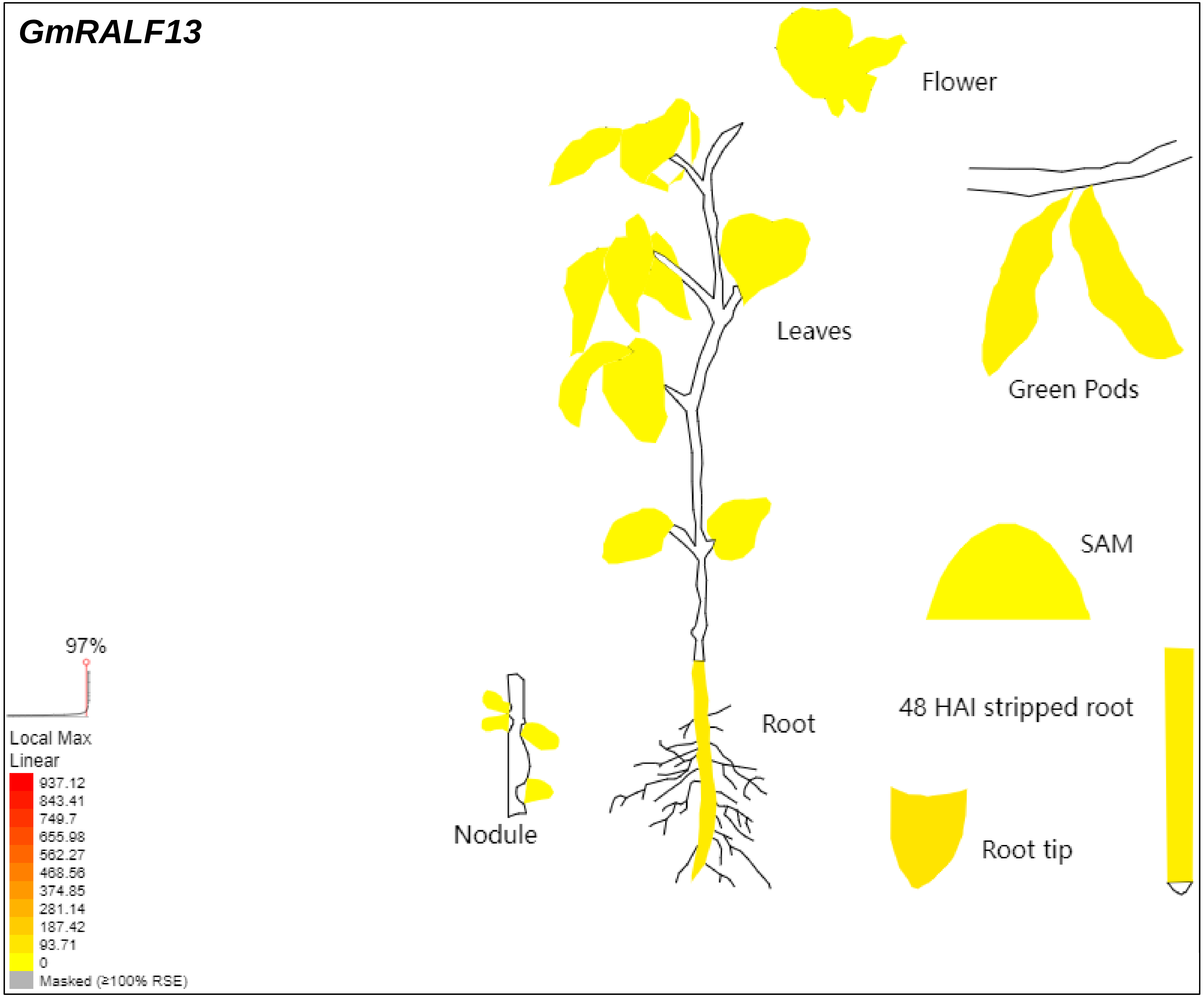
GmRALF13
Flower
Leaves
Green Pods
SAM
48 HAI stripped root
Root
Nodule
Root tip
97%
Local Max
Linear
937.12
843.41
749.7
655.98
562.27
468.56
374.85
281.14
187.42
93.71
0
Masked (≥100% RSE)

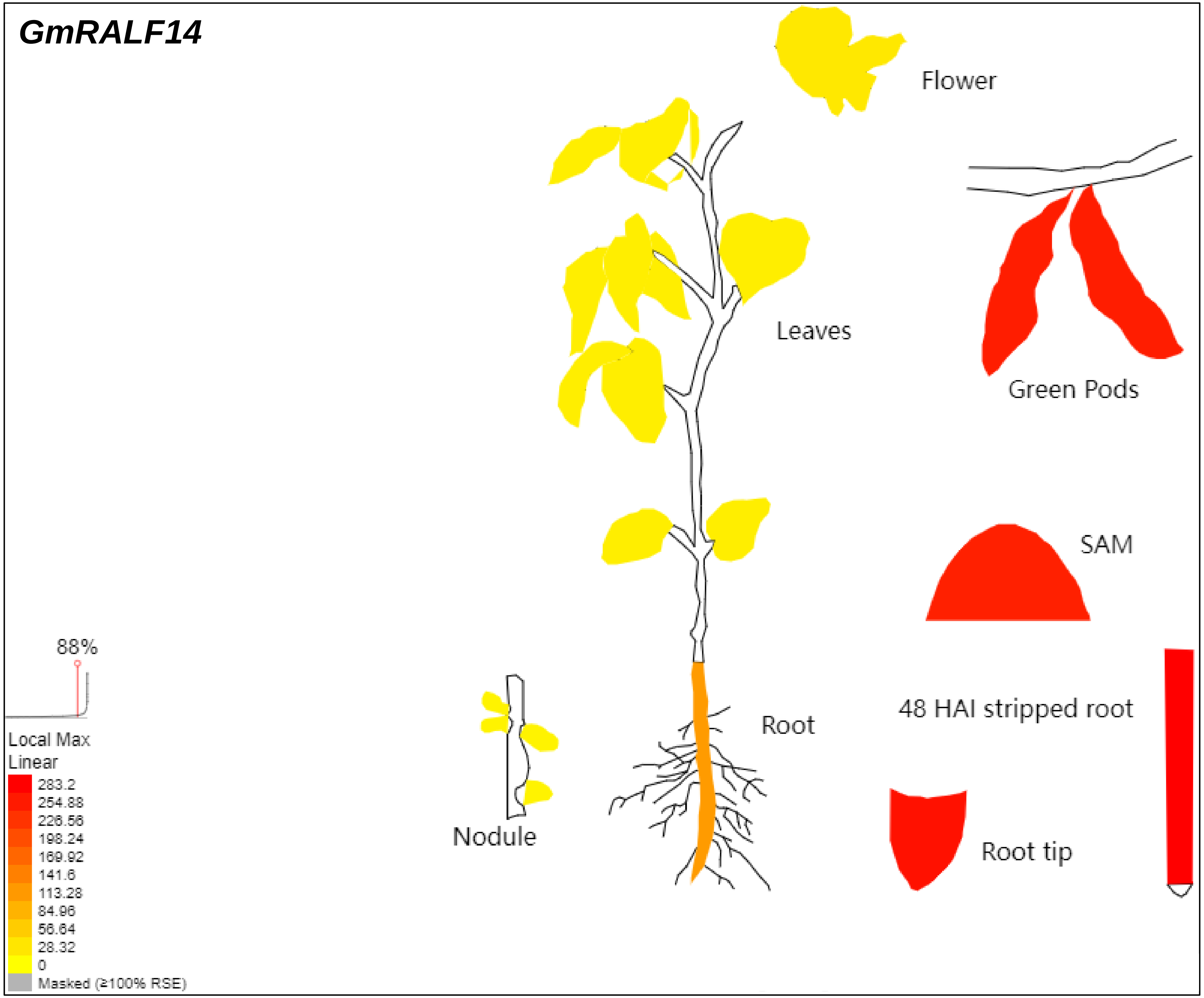

GmRALF14
Flower
Leaves
Green Pods
SAM
88%
Local Max
Linear
283.2
254.88
226.56
198.24
169.92
141.6
113.28
84.96
56.64
28.32
0
Masked (≥100% RSE)
Nodule
Root
48 HAI stripped root
Root tip

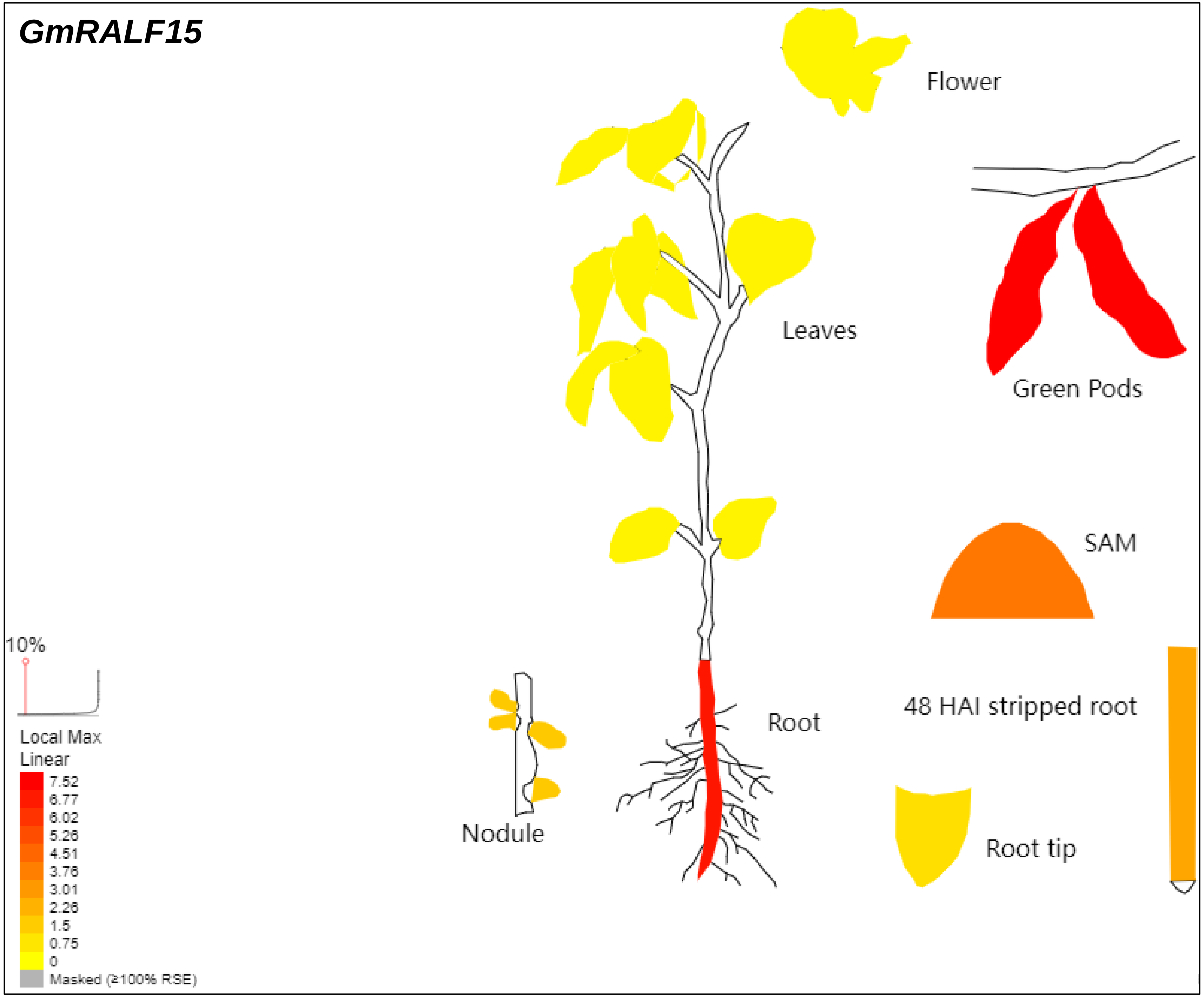

GmRALF15
Flower
Leaves
Green Pods
SAM
Root
48 HAI stripped root
Nodule
Root tip
10%
Local Max
Linear
7.52
6.77
6.02
5.26
4.51
3.76
3.01
2.26
1.5
0.75
0
Masked (≥100% RSE)

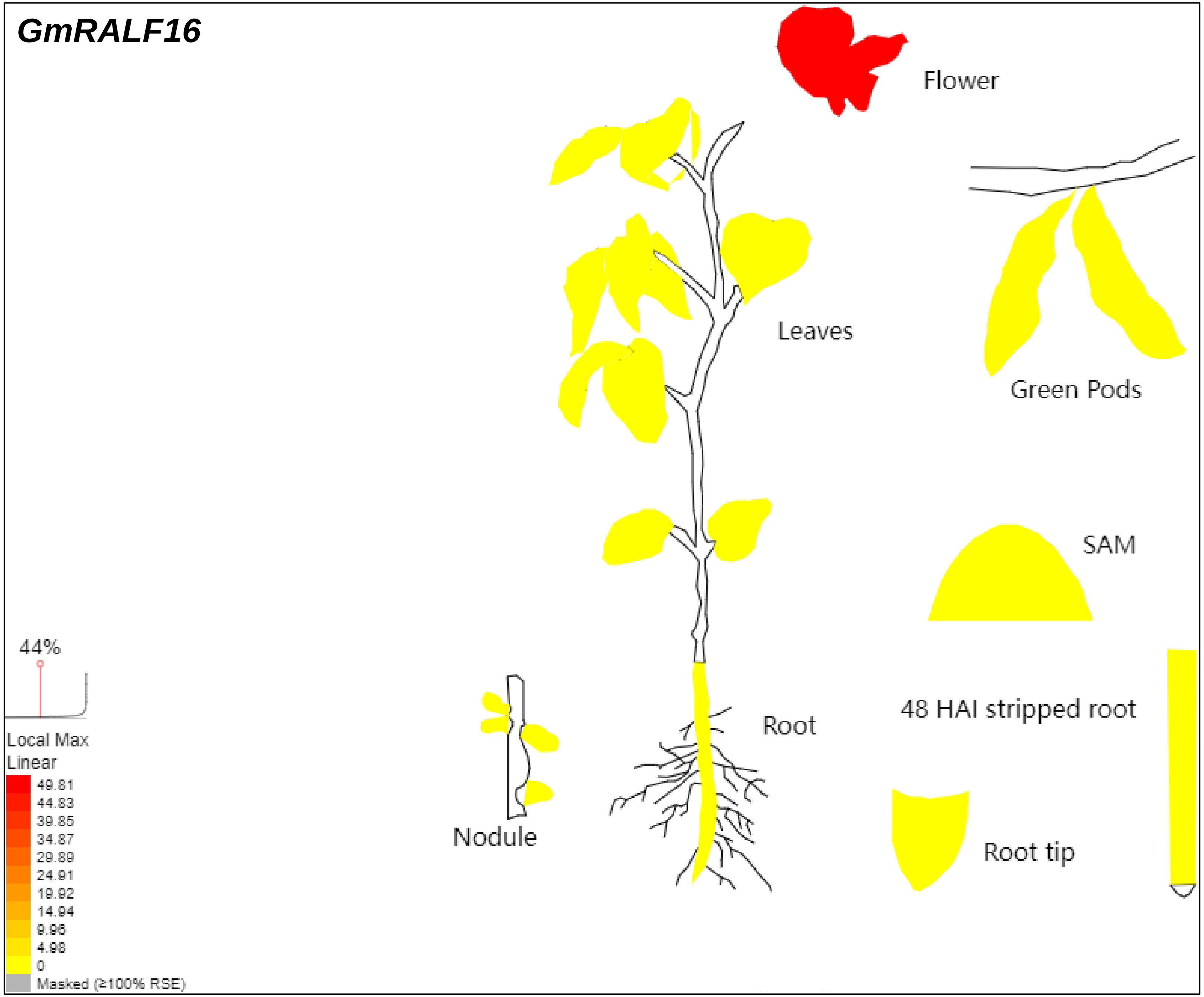

GmRALF16
Flower
Leaves
Green Pods
SAM
48 HAI stripped root
Root
Nodule
Root tip
44%
Local Max
Linear
49.81
44.83
39.85
34.87
29.89
24.91
19.92
14.94
9.96
4.98
0
Masked (≥100% RSE)

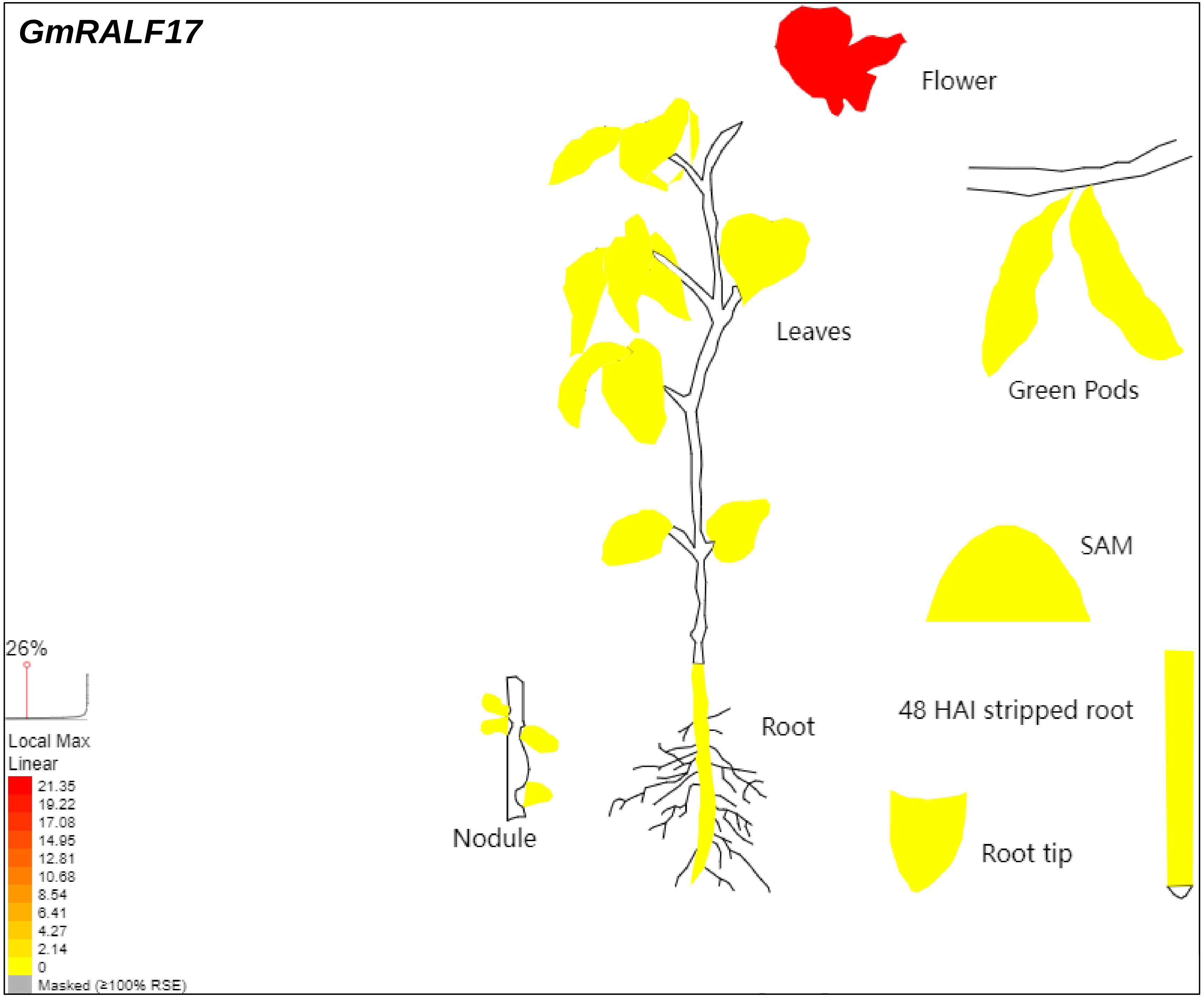
GmRALF17
Flower
Leaves
Green Pods
SAM
48 HAI stripped root
Root
Nodule
Root tip
26%
Local Max
Linear
21.35
19.22
17.08
14.95
12.81
10.68
8.54
6.41
4.27
2.14
0
Masked (≥100% RSE)

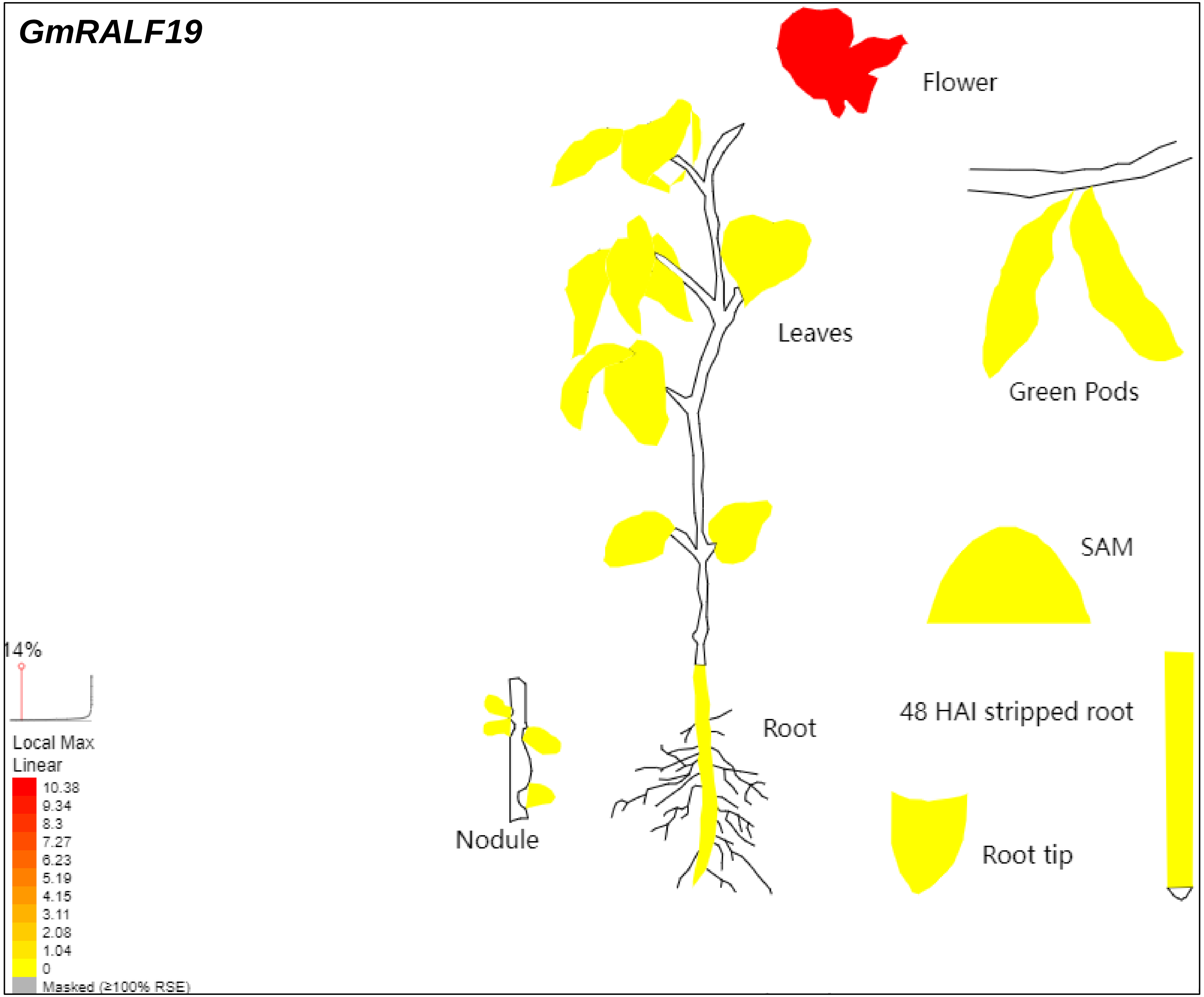
GmRALF19
Flower
Leaves
Green Pods
SAM
48 HAI stripped root
Root
Nodule
Root tip
14%
Local Max
Linear
10.38
9.34
8.3
7.27
6.23
5.19
4.15
3.11
2.08
1.04
0
Masked (≥100% RSE)

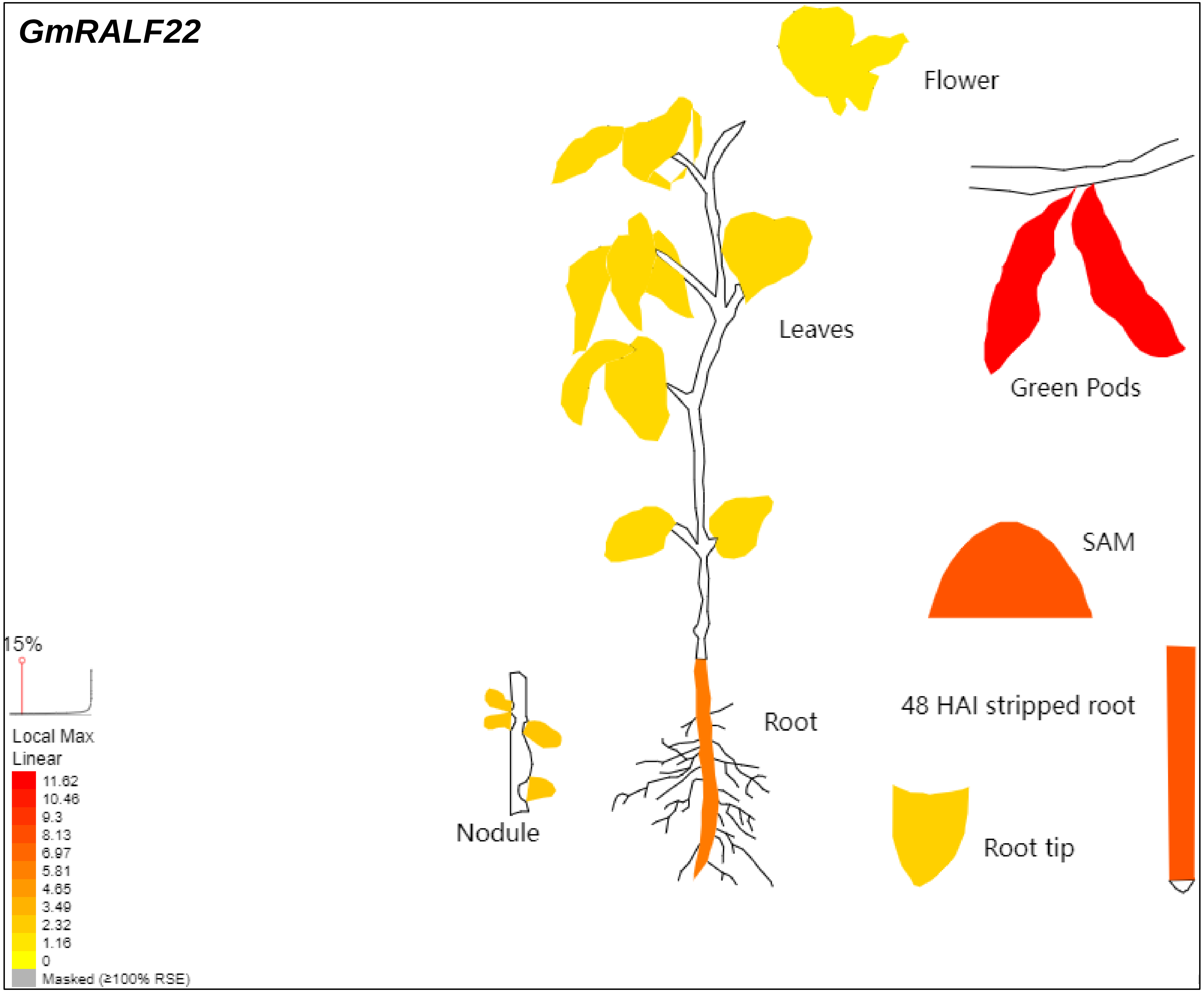

GmRALF22
Flower
Leaves
Green Pods
SAM
48 HAI stripped root
Root
Nodule
Root tip
15%
Local Max
Linear
11.62
10.46
9.3
8.13
6.97
5.81
4.65
3.49
2.32
1.16
0
Masked (≥100% RSE)

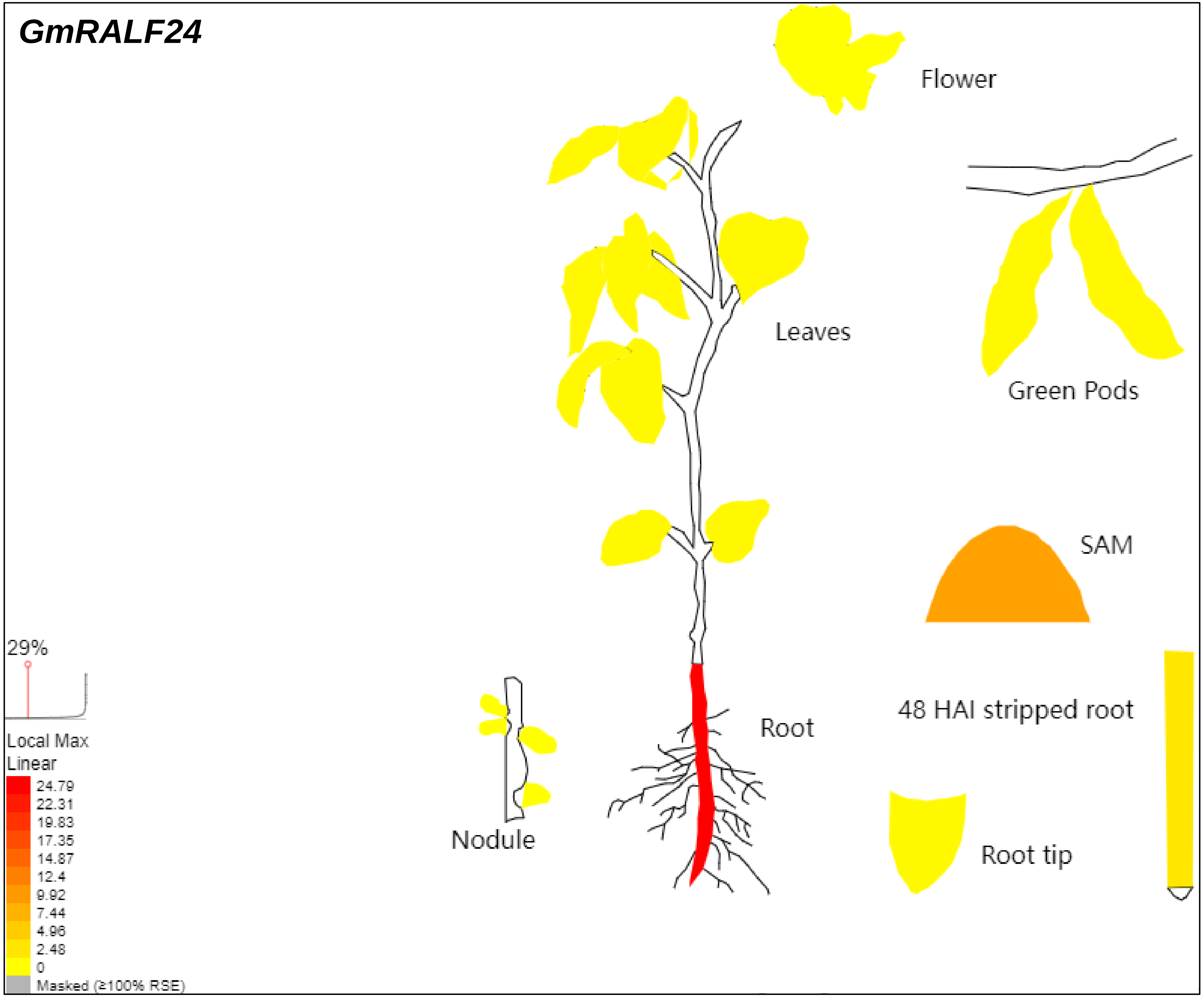
GmRALF24
Flower
Leaves
Green Pods
SAM
48 HAI stripped root
Root
Nodule
Root tip
29%
Local Max
Linear
24.79
22.31
19.83
17.35
14.87
12.4
9.92
7.44
4.96
2.48
0
Masked (≥100% RSE)

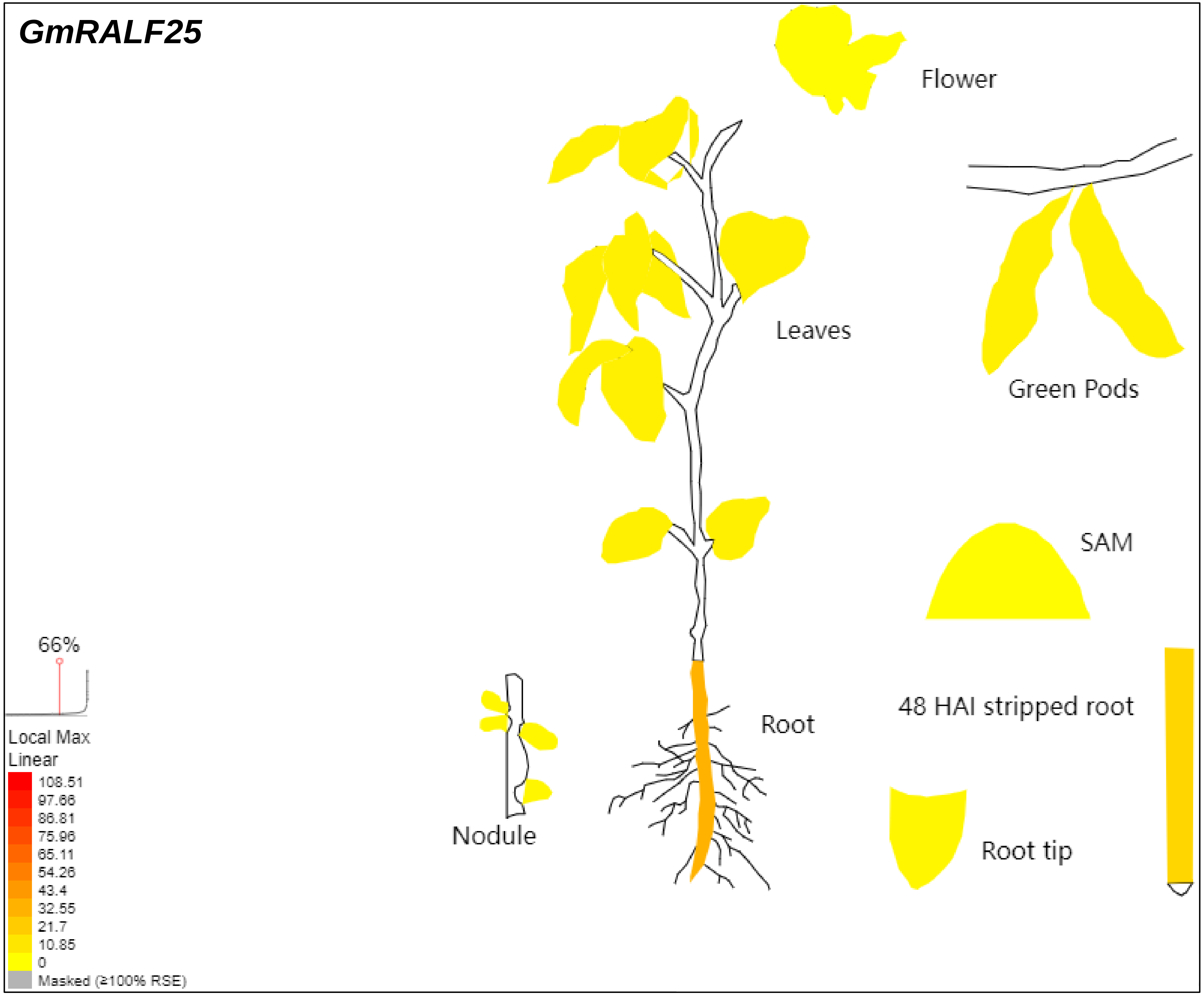
GmRALF25
Flower
Leaves
Green Pods
SAM
48 HAI stripped root
Root
Nodule
Root tip
66%
Local Max
Linear
108.51
97.66
86.81
75.96
65.11
54.26
43.4
32.55
21.7
10.85
0
Masked (≥100% RSE)

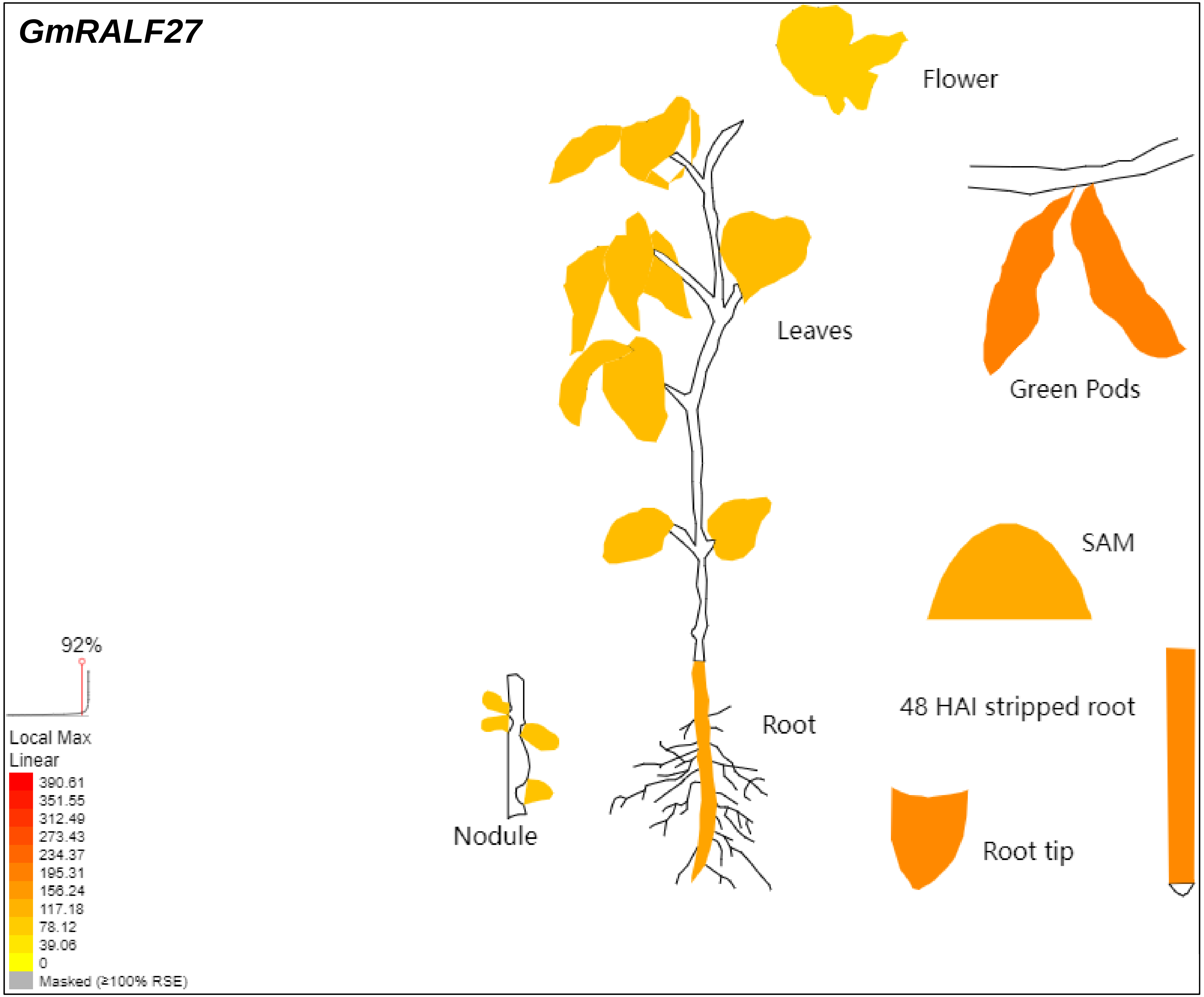
GmRALF27
Flower
Leaves
Green Pods
SAM
48 HAI stripped root
Root
Nodule
Root tip
92%
Local Max
Linear
390.61
351.55
312.49
273.43
234.37
195.31
156.24
117.18
78.12
39.06
0
Masked (≥100% RSE)
